# Disturbance of gut microbiota in diabetes related macroangiopathy: Evidence from the gut bacteriome and mycobiome

**DOI:** 10.1016/j.isci.2025.112856

**Published:** 2025-06-09

**Authors:** Xiaoling Gou, Yuqing Chen, Yi Zong, Xuemei Huang, Yihong Shen, Lijie Wang, Yifan Liu, Yuchi He, Jialong Jia, Xiyu Zhang, Sihan Peng, Xianhua Zhou, Ya Liu, Jing Zhang, Gang Fan

**Affiliations:** 1 State Key Laboratory of Southwestern Chinese Medicine Resources, School of Ethnic Medicine and Meishan Hospital, Chengdu University of Traditional Chinese Medicine, Chengdu 611137, P.R. China; 2 Department of Endocrinology, Hospital of Chengdu University of Traditional Chinese Medicine, Chengdu 610075, P.R. China

**Keywords:** Biological sciences, Microbiome

## Abstract

Diabetes related macroangiopathy (DMA) is a major complication of type 2 diabetes (T2D), impacting both morbidity and mortality. This study characterized the gut bacteriome and mycobiome in 179 adults, including 58 with DMA, 71 with T2D, and 50 healthy controls. The gut microbiome of DMA subjects exhibited reduced alpha diversity, and a distinct microbial composition compared with healthy control. Two bacterial families, six bacterial genera, and four bacterial species exhibited significant differences between DMA and T2D subjects. Additionally, in the mycobiome group, Xylariales was significantly decreased in DMA subjects compared with T2D subjects. Disruptions in transkingdom interactions between gut bacteria and fungi supported microbiota dysbiosis in DMA. A diagnostic model combining bacterial and fungal markers achieved an AUC of 94.20%. This work deepens our understanding of the microbial landscape associated with macroangiopathy in diabetes and highlights potential microbial targets for diagnostics and therapeutic intervention.

## Highlights

• The gut microbiota profile in diabetes related macroangiopathy (DMA) remains unclear
• DMA subjects show reduced microbial diversity and disrupted bacteria-fungi interactions
• Gut microbiota changes may serve as noninvasive biomarkers for DMA diagnosis and therapy

## Graphical abstract

**Figure:**
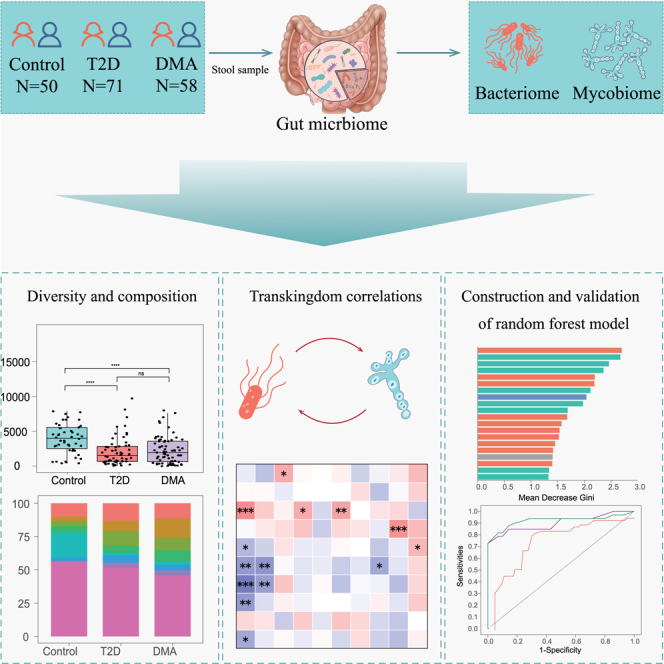

## Introduction

Diabetes related macroangiopathy (DMA) represents a prevalent complication of type 2 diabetes (T2D), afflicting approximately 30% of diabetic patients. These complications encompass ischemic heart disease, cerebrovascular disorders, and peripheral arterial disease, characterized by features such as intraplaque neovascularization, augmented vascular permeability, tissue edema, and cardiac microvascular dysfunction,^1^ leading to frequent hemorrhage and rupture of atherosclerotic plaques.^2^ Such manifestations profoundly impact patients’ quality of life and survival. DMA is intricately associated with various physiological disruptions, including endothelial dysfunction, heightened oxidative stress, impaired lipid metabolism, mitochondrial dysfunction, and accumulation of advanced glycation end-products (AGEs).^3^^,^^4^

Bacteria and fungi are critical constituents of the human gut microbiome.^5^ Gut bacteria and fungi interact in complex ways, such as competition, symbiosis, and co-metabolism, to collectively shape and influence the gut microenvironment.^6^ In recent years, accumulating evidence has underscored the pivotal role of gut bacteria in shaping diseases such as nonalcoholic fatty liver disease (NAFLD), diabetes, and inflammatory bowel disease (IBD).^7^^,^^8^^,^^9^^,^^10^ Gut bacteria are involved in the synthesis of small molecule metabolites, such as short-chain fatty acids (SCFAs) and bile acids, which influence the development of diseases through various signaling pathways.^11^^,^^12^ Moreover, recent studies have indicated that fungi also play an important role in the occurrence of chronic diseases. For example, alterations in the gut fungal community have been associated with several complex diseases such as fatty liver, diabetes, and cancer.^13^^,^^14^ Fungi can influence metabolic diseases through inducing immune responses, fungal-bacterial interactions, and producing biologically active metabolites.^15^

In recent years, studies have indicated significant differences in the microbiota of patients with T2D compared to healthy controls, characterized by a reduction in beneficial bacteria and an increase in potentially pathogenic or conditional pathogenic organism.^16^^,^^17^ Furthermore, interactions among gut microbiota have been closely linked to diabetic nephropathy.^10^^,^^18^ However, the composition and interactions of gut microbiota in DMA are still unclear. Therefore, this study aims to characterize differences in the gut microbiota composition among patients with DMA, including bacteria and fungi. Additionally, the study explores the potential association between these differences and the occurrence of DMA. These findings are poised to deepen our comprehension of the pivotal role played by the gut microbiota in the pathogenesis of DMA.

## Results

### Clinical characteristics of the study population

A total of 179 participants were enrolled, with approximately equal representation from both sexes. Significant differences in age were observed among the three groups, with individuals in the DMA subjects having a higher median age (54.0 years). In comparison to healthy controls, participants in the T2D and DMA subjects exhibited significantly elevated levels of fasting glucose, systolic blood pressure (SBP), diastolic blood pressure (DBP), and triglycerides (TG), along with decreased levels of high-density lipoprotein cholesterol (HDL-C). Additionally, individuals with DMA demonstrated significantly higher SBP and DBP levels compared to those with T2D. The median SBP and DBP values in DMA patients were 152.00 mmHg and 93.00 mmHg, respectively, suggesting concurrent hypertension.

### Diversity and compositional alterations of gut bacteriome in DMA

Bacterial 16S rRNA sequencing was performed to evaluate differences in the gut bacteriome among the three groups. On average, 13,497,806 clean reads were obtained from the 16S rRNA sequencing. Table S1 details the sequencing reads for each sample. Initially, we compared bacterial alpha diversity among the three groups.

**Figure 1:**
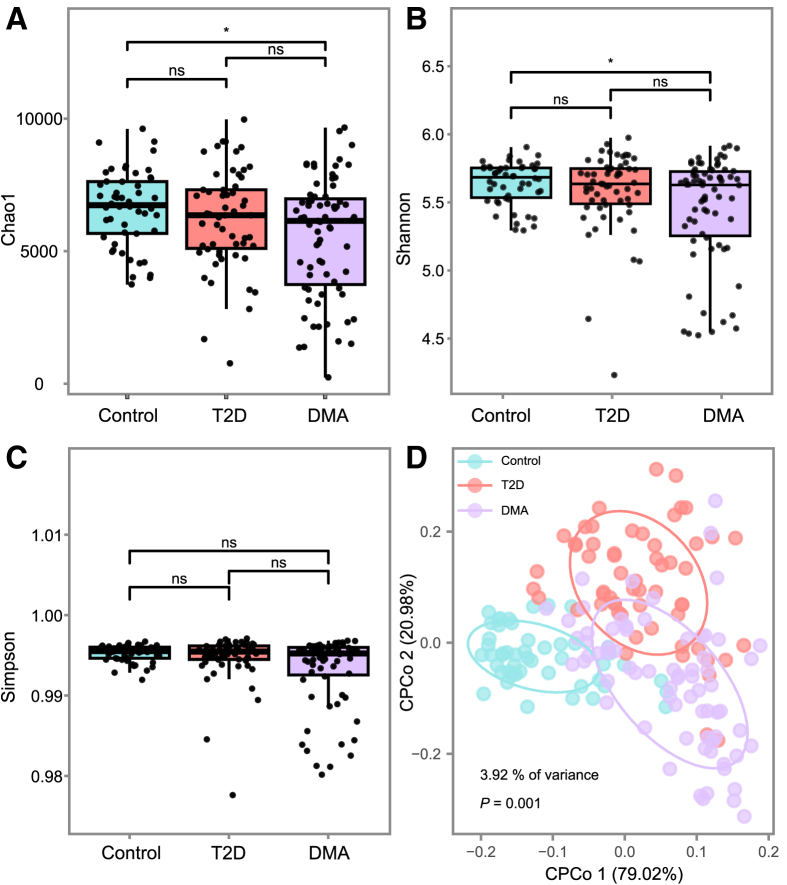
Alterations of the gut bacteria in DMA compared with T2D and healthy controls (A–C) Differences between the 3 groups in bacteria richness based on the (A) Chao1 index and bacteria diversity based on the (B) Shannon and (C) Simpson indices at the contig level. For the box plots, the boxes extend from the first to the third quartile (25th to 75th percentiles), with the center line indicating the median. (D) CPCoA analysis based on Bray-Curtis distance between DMA, T2D and healthy controls. *p* < 0.05 was considered statistically significant. ^∗^*p* < 0.05, ^∗∗^*p* < 0.01 and ^∗∗∗^*p* < 0.001.

When comparing patients with T2D to the normal healthy controls, there were no significant differences observed in the richness and diversity of bacteria. While patients with DMA showed significantly lower bacterial diversity (as measured by the Shannon, Chao1, and Richness indices) compared to healthy controls at the contig level (Figures 1A–1C). Additionally, we assessed the variations in bacterial communities (beta diversity) across the three study groups using CPCoA analysis based on the Bray-Curtis distance metric. The results showed that T2D patients, DMA patients, and healthy controls were separated into three distinct clusters (*p* = 0.001; Figure 1D). These findings suggest significant differences in the gut bacterial profiles of patients with DMA compared to those of T2D patients and healthy controls.

We further examined the relationship between age, sex, obesity, and several commonly prescribed medications and gut bacterial alpha diversity. Notably, there was no significant correlation between age and the three alpha diversity indices (Figure S1). Likewise, there were no significant differences in the three indices between obese and lean individuals. Moreover, there were no significant differences in bacterial diversity (as indicated by the Shannon and Simpson indices) or richness (measured by the Chao1 index) between males and females across the three groups (Figure S1). Metformin, statins, NSAIDs, sulfonylureas, and ARBs are commonly prescribed medications for patients in both the T2D and DMA subjects. Our analysis showed no significant difference in bacterial diversity between patients receiving these drugs and those who were not (Figure S2). These findings collectively indicate that age, sex, obesity, and commonly prescribed medications have no significant influence on gut bacterial diversity in patients with DMA.

We conducted a further analysis of the compositional differences in gut bacteria among the three groups using MaAsLin2. Clostridiales was the predominant bacterial order. A significant decrease in Bacteroidales, Selenomonadales, and Pasteurellales was observed in T2D subjects compared to healthy controls (Figure S3C). Furthermore, compared to healthy controls, DMA subjects exhibited a significant increase in eight orders (including Enterobacteriales, Bifidobacteriales, and Lactobacillales) and a notable decrease in four orders (including Bacteroidales, Selenomonadales, and Pasteurellales) (Table S2).

Lachnospiraceae was the predominant bacterial family across all three groups on average. Compared to healthy controls, T2D subjects exhibited a significant increase in 21 families and a significant decrease in 10 families (Table S3). Similarly, compared to healthy controls, DMA subjects exhibited a significant increase in 27 families (including Enterobacteriaceae, Bifidobacteriaceae, and Lactobacillaceae) and a significant decrease in 15 families (including Bacteroidaceae, Prevotellaceae, and Veillonellaceae) (Table S3). Notably, two bacterial families exhibited significant differences between DMA and T2D subjects: Succinivibrionaceae was significantly increased, whereas Planococcaceae was significantly decreased in DMA subjects compared to T2D subjects (Figure S3D).

**Figure 2:**
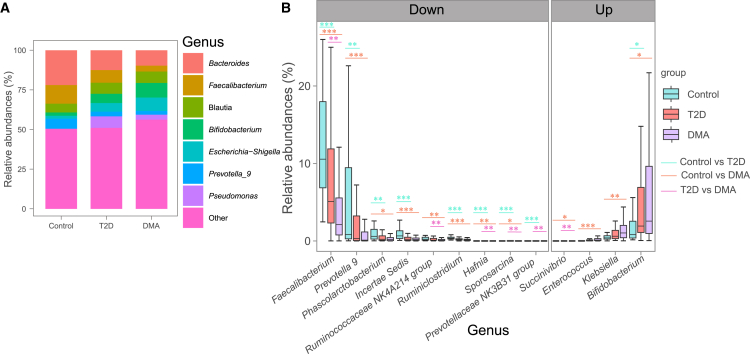
Differential bacterial genera in DMA compared with T2D and healthy controls (A) Stacked bar chart showing bacterial composition at the genus level. (B) Differential bacterial genera in DMA compared with healthy T2D and controls were determined by MaAsLin2 analysis and adjusted for confounders, including age, sex, BMI, SBP, and DBP. *p* < 0.05 was considered statistically significant. ^∗^*p* < 0.05, ^∗∗^*p* < 0.01 and ^∗∗∗^*p* < 0.001. For the box plots, the boxes extend from the first to the third quartile (25th to 75th percentiles), with the center line indicating the median.

At the genus level, compared to healthy controls, T2D subjects exhibited a significant increase in 52 genera and a significant decrease in 43 genera (Table S4). Similarly, compared to healthy controls, DMA subjects exhibited a significant increase in 43 genera (including *Bifidobacterium*, *Klebsiella*, and *Enterococcus*) and a significant decrease in 49 genera (including *Faecalibacterium*, *Prevotella 9*, and *Phascolarctobacterium*) (Figure 2; Table S4). Notably, six bacterial genera exhibited significant differences between DMA and T2D subjects: *Faecalibacterium*, *Ruminococcaceae_NK4A214_group*, *Hafnia*, *Prevotellaceae_NK3B31_group*, and *Sporosarcina* were significantly decreased, whereas *Succinivibrio* was significantly increased in DMA subjects compared to T2D subjects.

At the species level, 36 bacterial species exhibited significant alterations in T2D subjects compared to healthy controls. Moreover, 31 bacterial species exhibited notable differences in DMA subjects compared to healthy controls (Table S5). Notably, *Parabacteroides distasonis*, *Hafnia alvei*, and *Sporosarcina newyorkensis* exhibited a significant decrease in DMA subjects compared to T2D subjects. Conversely, *Lactobacillus acidophilus* exhibited a significant increase in DMA subjects compared to T2D subjects (Figure S3E). Taken together, these results suggest that patients with DMA experience significant gut bacterial dysbiosis, characterized by alterations in both diversity and taxonomy.

### Diversity and compositional alterations of gut mycobiome in DMA

**Figure 3:**
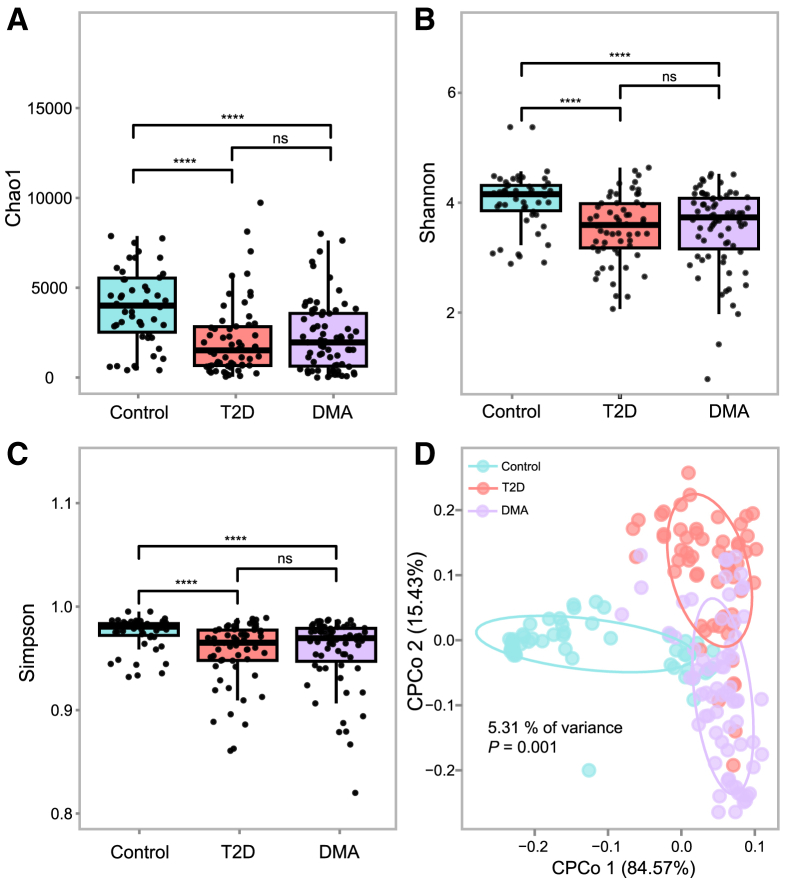
Alterations of the gut fungi in DMA compared with T2D and healthy controls (A–C) Differences between the 3 groups in fungi richness based on the (A) Chao1 index and fungi diversity based on the (B) Shannon and (C) Simpson indices at the contig level. For the boxplots, the boxes extend from the first to the third quartile (25th to 75th percentiles), with the center line indicating the median. (D) CPCoA analysis based on Bray-Curtis distance between DMA and T2D compared with healthy controls. *p* < 0.05 was considered statistically significant. ^∗^*p* < 0.05, ^∗∗^*p* < 0.01 and ^∗∗∗^*p* < 0.001.

The sequencing effort targeting the ITS region yielded an average of 125,300 clean reads Table S6 details the sequencing reads for each sample. Initially, we compared alpha diversity across three groups, revealing significantly lower fungal diversity (as indicated by Shannon indices) and richness (as measured by the Chao1 and Richness index) in patients with DMA compared to healthy controls (Figure 3). These findings suggest an altered overall composition of the gut mycobiome in the DMA subjects compared to healthy controls. Furthermore, employing CPCoA based on Bray-Curtis distance, we assessed differences in gut mycobiome communities (beta diversity) among healthy controls, T2D patients and DMA patients. The results demonstrated segregation of T2D patients, DMA patients, and healthy controls into three distinct clusters (*p* = 0.001; Figure 3D). These data indicate notable differences in gut microbiota profiles between DMA patients and both healthy controls as well as T2D patients.

Additionally, we investigated the association of age, sex, obesity, and several commonly used drugs with gut fungal alpha diversity. There was no significant correlation observed between age and all three alpha diversity indices across the three groups (Figure S4A). Similarly, no significant differences were observed for the three indices between males and females across the three groups (Figure S4B). Additionally, no significant difference in fungal diversity and richness was observed between obese and lean individuals across the three groups (Figure S4C).

Likewise, metformin, statins, non-steroidal anti-inflammatory drugs (NSAIDs), sulfonylureas, and angiotensin receptor blockers (ARBs) are commonly prescribed medications for patients with T2D and DMA. No significant difference in fungal diversity was observed between patients taking these drugs and those not taking them (Figure S5). In summary, age, sex, obesity, and common medications have minimal impact on the gut mycobiome in patients with T2D and DMA.

**Figure 4:**
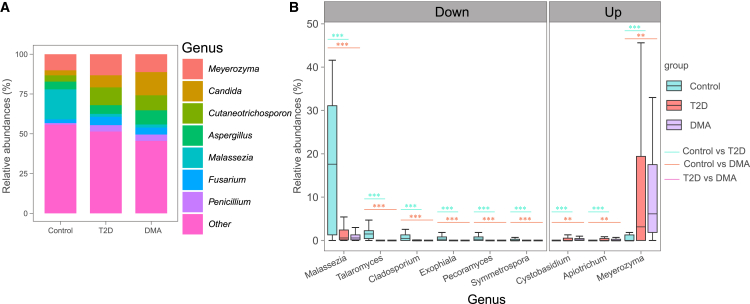
Differential fungal genera in DMA compared with T2D and healthy controls (A) Stacked bar chart showing fungal composition at the genus level. (B) Differential fungal genera in DMA compared with healthy T2D and controls were determined by MaAsLin2 analysis and adjusted for confounders, including age, sex, BMI, SBP, and DBP. *q* < 0.05 was considered statistically significant. ^∗^*q* < 0.05, ^∗∗^*q* < 0.01 and ^∗∗∗^*q* < 0.001. For the boxplots, the boxes extend from the first to the third quartile (25th to 75th percentiles), with the center line indicating the median.

We employed MaAsLin2 analysis to identify gut mycobiome signatures associated with complications of DMA. At the order level, Malasseziales and Saccharomycetales were the predominant fungal orders, with 14 distinct fungal orders exhibiting significant differences between DMA subjects and healthy controls (Figure S6C; Table S7). Moreover, Xylariales was significantly decreased in DMA subjects compared with T2D subjects (Figure S6C). At the family level, *Malasseziaceae* was the predominant fungal family. A total of 18 fungal families exhibited significant differences between DMA subjects and healthy controls, while 14 fungal families exhibited significant differences between T2D subjects and healthy control (Figure S6D; Table S8). At the genus level, 24 fungal genera exhibited significant differences between DMA subjects and healthy controls, including *Malassezia*, *Meyerozyma*, and *Cystobasidium*. Similarly, 25 fungal genera exhibited significant differences between T2D subjects and healthy controls, including *Talaromyces*, *Meyerozyma*, and *Penicillium* (Figure 4; Table S9). At the species level, 15 fungal species exhibited significant alterations in DMA subjects compared to healthy controls (Figure S6E; Table S10). 14 fungal species exhibited significant alterations in T2D subjects compared to healthy controls (Figure S6E; Table S10).

### Transkingdom correlations between gut bacteriome and mycobiome are disrupted in DMA

**Figure 5:**
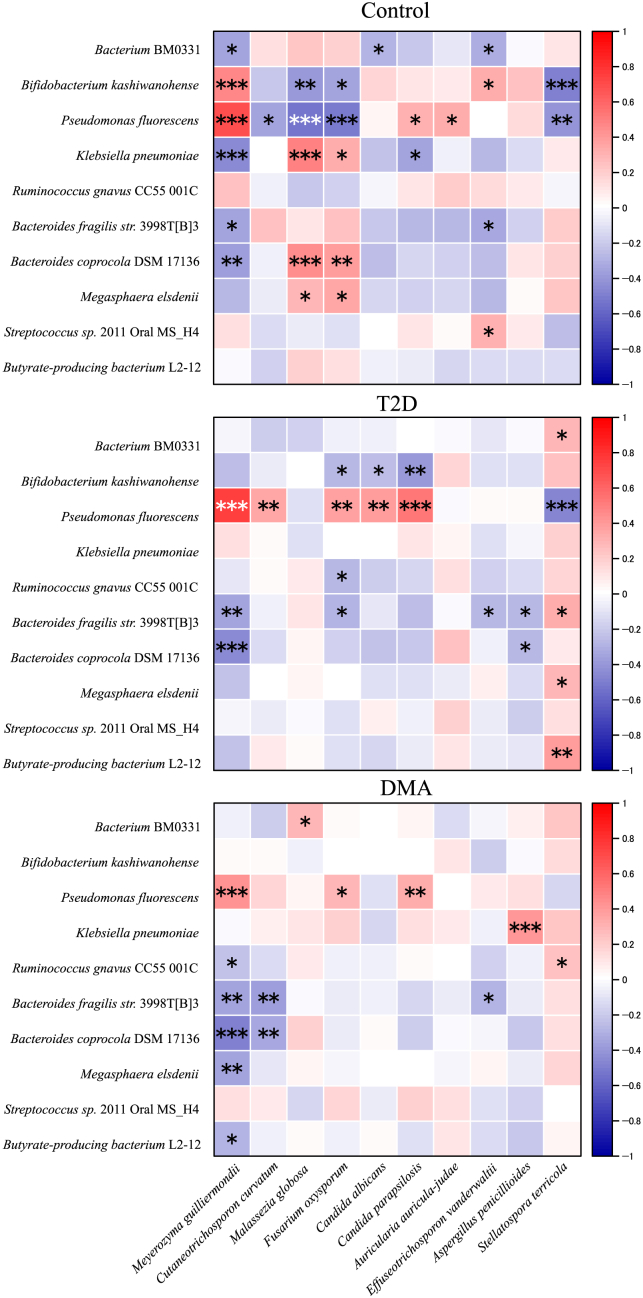
Alterations of transkingdom correlations between the gut bacteriome and mycobiome in DMA compared with T2D and healthy controls Heatmap shows color-coded Spearman’s correlations of the most abundant 10 bacteria genus with the most abundant 10 fungal genus. Red color indicates positive correlation and blue color indicates negative correlation. Significant correlations are displayed with an asterisk. ^∗^*p* < 0.05, ^∗∗^*p* < 0.01 and ^∗∗∗^*p* < 0.001.

Transkingdom correlations between gut bacteria and fungi play crucial roles in maintaining health and contributing to disease states. We analyzed the correlation between fungal and bacterial species. Notably, a decrease in both positive and negative correlations between the bacteriome and mycobiome was detected in the T2D subjects compared to the healthy controls. Moreover, significantly fewer correlations between the bacteriome and mycobiome were observed in the DMA subjects compared to the healthy controls (12 vs. 27; χ2 test, *p* < 0.001). Likewise, fewer positive and negative correlations between the bacteriome and mycobiome were evident in the DMA subjects compared to the T2D subjects (Figure 5). These changes in bacteria-fungi correlations were primarily due to the loss of certain associations and the emergence of novel ones. For example, novel correlation associations emerged between *Aspergillus penicillioides* and several bacteria in both T2D and DMA subjects compared to healthy controls. Moreover, all the correlations between *Candida albicans* and bacteria present in healthy controls and T2D patients were absent in the DMA subjects. Likewise, all the correlations between *Bifidobacterium kashiwanohense* and fungi present in healthy controls and T2D patients were absent in the DMA subjects. Taken together, these findings indicate that robust bacterial-fungi interactions observed in healthy controls are disrupted in DMA patients.

### The combination of bacteriome and mycobiome markers enhances diagnostic performance for DMA

**Figure 6:**
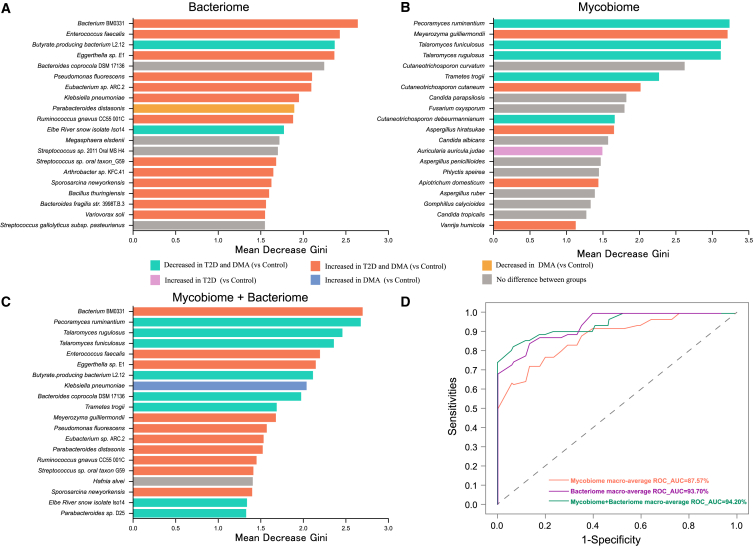
The combination of gut bacteriome and mycobiome has good potential in diagnosing between DMA, T2D, and healthy controls based on the Random Forest model (A–C) The important features for (A) bacteriome (top 20), (B) mycobiome (top 20), and (C) combined bacteriome and mycobiome (top 20). The color of the bars corresponds to differential abundance between groups. (D) The ROC curve analysis for each of the three models based on macro-average, including their AUC values.

To assess the potential of gut microbiota as diagnostic markers for DMA, we developed a machine learning classifier using the random forest (RF) algorithm. The aim was to pinpoint gut microbial markers capable of distinguishing among T2D, DMA, and healthy controls. Our analysis revealed a mycobiome taxa signature of 51 species as the most effective marker set across the three groups, achieving an area under the curve (AUC) of 87.57% (Figure 6A; Table S11). Notably, *Pecoramyces ruminantium* emerged as the most influential feature, as evidenced by the mean decrease Gini, followed by *Meyerozyma guilliermondii* and *Talaromyces funiculosus*. Additionally, the bacteriome model (AUC = 93.70%) exhibited superior predictive capability compared to the mycobiome model (Figure 6B; Table S12). Notably, *Bacterium* BM0331 emerged as the most influential feature, as indicated by the mean decrease Gini, followed by *Enterococcus faecalis* and *Butyrate*-producing bacterium L2-12. Additionally, integrating the bacteriome and mycobiome yielded an AUC of 94.20% in distinguishing the three groups (Figures 6C and S7; Table S13). Similarly, *Bacterium* BM0331 continued to demonstrate the greatest impact, as indicated by the mean decrease Gini, followed by *Pecoramyces ruminantium* and *Talaromyces rugulosus*. In summary, our findings indicate that integrated bacteriome and mycobiome markers hold promise for distinguishing DMA from T2D and healthy controls.

## Discussion

The association between gut microbiota and T2D is well-established, whereas the correlation between gut microbiota and DMA remains elusive.^10^ In this pioneering investigation, we identified substantial alterations in the gut microbiota of participants with DMA using analyses of bacterial 16S rRNA gene sequences and internal transcribed spacer (ITS) sequencing. We found that DMA, a significant complication of T2D, displayed notable dysbiosis in gut microbiota in this study. These results raise an intriguing question about the potential role of gut microbiota in DMA.

In this study, we identified six significantly altered bacterial genera in individuals with DMA compared to T2D subjects. These genera, including *Faecalibacterium*, *Hafnia*, *Prevotellaceae NK3B31 group*, *Ruminococcaceae NK4A214 group*, *Sporosarcina*, and *Succinivibrio*, are closely associated with the development of DMA. Additionally, we identified four significantly altered bacterial species in individuals with DMA compared to T2D subjects. These species, including *P. distasonis*, *H. alvei*, *S. newyorkensis*, and *L. acidophilus*, are closely associated with the development of DMA. *Faecalibacterium* represents a class of probiotic bacteria commonly found in the human gut.^19^ Butyrate, a key metabolite produced by *Faecalibacterium*.^20^ potentially enhances insulin sensitivity through various mechanisms.^21^ Moreover, *Faecalibacterium* might mitigate diabetic macrovascular lesions by modulating intestinal cholesterol metabolism, consequently reducing blood low-density lipoprotein cholesterol (LDL-C) levels.^22^^,^^23^ *P. distasonis* is the type species of the newly established *Parabacteroides* genus and a gut commensal belonging to the phylum Bacteroidetes.^24^ A recent study demonstrated that *P. distasonis* alleviates obesity and metabolic dysfunction by producing succinate and secondary bile acids.^25^ Furthermore, *P. distasonis* and other intestinal microbiota convert chenodeoxycholic acid (CDCA) into ursodeoxycholic acid (UDCA) and lithocholic acid (LCA).^26^ Similarly, this study detected significant alterations in gut fungal composition among individuals with DMA, identifying specific fungal genera linked to the condition. A total of 24 fungal genera demonstrated significant differences between DMA and healthy controls, including *Malassezia*, *Meyerozyma*, and *Cystobasidium*. Notably, the relative abundance of Xylariales was significantly reduced in individuals with DMA compared to those with T2D. Previous studies indicate that fungi belonging to the order Xylariales produce antimicrobial compounds, such as nodulisporic acids and sordarin.^27^

The human gut microbiota comprises a vast and complex community of microorganisms, including bacteria and fungi. The dysbiosis of the interactions of bacteria and fungi is closely associated with various disease states.^28^ Our findings reveal a disrupted interplay between bacteria and fungi in DMA patients compared to T2D and healthy controls. Specifically, these transkingdom correlations were significantly disrupted in cases of T2D and DMA, characterized by alterations in both negative and positive correlations. Notably, certain bacteria, such as *Ruminococcus gnavus* CC55 001C demonstrated significant correlations with various fungi in individuals with T2D and DMA compared with healthy controls. Furthermore, the associations between the diabetes-related pathogenic bacterium *Klebsiella pneumoniae* and fungi, which were observed in healthy subjects, diminished in those with T2D and DMA. In addition, the number of negative and positive correlations between bacteria and fungi were all reduced in the DMA subjects compared to the T2D subjects. The substantial alterations in transkingdom correlations between gut mycobiome and bacteriome potentially indicate a disturbance within the entire ecosystem in DMA. Emphasizing the potential influence of maintaining a harmonious symbiosis between gut bacteria and fungi is essential for maintaining intestinal microecological balance.

In this study, an RF classification model was utilized to investigate the potential of gut microbiota as diagnostic markers for DMA. As an ensemble learning method, RF possesses excellent anti-overfitting capabilities, variable importance assessment, and classification performance.^29^^,^^30^ It is effective in handling high-dimensional feature spaces and capturing nonlinear relationships when dealing with complex and highly heterogeneous gut microbiota data.^30^ By integrating multiple decision trees, it reduces the uncertainty of individual models.^31^ Previous research has highlighted the potential of gut bacterial markers in diagnosing T2D.^32^^,^^33^ Attention should be directed toward DMA, given their elevated disability and mortality rates compared to diabetes alone.

Here, we demonstrate the utility of specific gut microbiota as biomarkers for distinguishing between patients with DMA, T2D, and healthy controls. The fungal markers exhibited good diagnostic performance, achieving an AUC of 87.57%. Moreover, the bacterial markers exhibited superior diagnostic performance, achieving an AUC of 93.70%. These biomarkers have the potential to predict complications associated with DMA. The gut microbiota does not exist in isolation, but rather forms a complex ecosystem, where the overall microbial community is crucial for diagnosing DMA. In our study, the combination of gut bacteria and fungi also demonstrated considerable capability in distinguishing between T2D, DMA, and healthy controls, achieving an AUC of 94.20%. Overall, our findings suggest that gut microbiota holds promise for non-invasively diagnosing DMA.

### Limitations of the study

Although our research has made important findings, some limitations should be acknowledged. First, the study used a cross-sectional design. This limits the ability to determine a causal relationship between gut microbiota alterations and the progression of DMA. Longitudinal studies are needed to clarify the role of gut microbiota in DMA pathogenesis. Second, we adjusted for age, sex, and hypertension when analyzing microbial differences. However, other factors, such as dyslipidemia and disease duration, may also affect the gut microbiota. These factors should be better controlled in future research. Third, only fasting blood glucose levels were used to assess the status of diabetes, and long-term blood glucose indicators, such as glycated hemoglobin (HbA1c), were lacking. Besides, as shown in Table 1, the median blood glucose level in the DMA group was slightly lower than in the T2D group. This might imply that the blood glucose control in the DMA group was better. However, the reasons behind it remain unclear and warrant further investigation.

**Table 1:** Clinical characteristics of the study cohort

Variables	Control (*n* = 50)	T2D (*n* = 58)	DMA (*n* = 71)	*p* value
Age, years	51.50 (46.00–55.25)	51.00 (45.75–57.00)	54.00 (50.00–57.00)	0.039^b^
Female, n (%)	31 (57.41)	27 (42.18)	30 (40.54)	0.129
Body mass index, kg/m^2^	24.30 (22.70–27.25)	24.02 (21.86–27.62)	24.84 (21.64–27.91)	0.902
Fasting glucose, mmol/L	4.83 (4.35–5.57)	6.79 (5.37–7.72)	5.93 (4.94–7.71)	<0.001^a^^,^^b^
TC, mmol/L	4.73 (4.32–5.20)	5.08 (4.14–5.84)	5.17 (4.16–6.25)	0.136
TG, mmol/L	1.36 (1.03–1.85)	1.83 (1.28–2.75)	2.01 (1.34–4.23)	<0.001^a^^,^^b^
HDL-C, mmol/L	1.36 (1.20–1.69)	1.15 (1.00–1.35)	1.11 (1.00–1.30)	<0.001^a^^,^^b^
SBP, mmHg	118.00 (111.75–128.25)	135.00 (125.00–146.25)	152.00 (137.00–165.00)	<0.001^a^^,^^b^^,^^c^
DBP, mmHg	72.50 (66.00–80.00)	80.00 (80.00–91.00)	93.00 (85.00–107.00)	<0.001^a^^,^^b^^,^^c^

Values are presented as median (interquartile range) for continuous variables or number (percentage) for categorical variables. Three groups were compared using one-way ANOVA with the Tukey post-hoc test for normally distributed variables or Kruskal-Wallis test with the Dunn post-hoc test for non-normally distributed variables. Categorical variables were compared by the χ^2^ test.

TC, total cholesterol; TG, triglycerides; HDL-C, high-density lipoprotein cholesterol; SBP, systolic blood pressure; DBP, diastolic blood pressure.

a *p* < 0.05 for T2D vs. healthy controls.

b *p* < 0.05 for DMA vs. healthy controls.

c *p* < 0.05 for DMA vs. T2D.

### Conclusion

This study provides evidence that DMA subjects are characterized by disturbances in gut microbiota diversity and taxonomic composition compared with healthy controls and T2D subjects. The significant differences observed suggest that gut microbiota may play an important role in the development of DMA. The gut microbiota markers have great potential for non-invasive diagnosis of DMA. Although more clinical validation and mechanistic studies are needed, this study contributes to our understanding of the role of the gut microbiome in the pathogenesis of DMA, and paves avenues for the development of diagnostic tools and therapeutic strategies.

## Resource availability

### Lead contact

Further information and requests for resources and reagents should be directed to and will be fulfilled by the lead contact, Gang Fan (fangang1111@163.com).

### Materials availability

This study did not generate new unique reagents.

### Data and code availability

• Data: All sequence files are available from the National Center for Biotechnology Information through BioProject: PRJNA1106213 (https://www.ncbi.nlm.nih.gov/bioproject/PRJNA1106213). The data sets generated during the current study are available from the corresponding author upon reasonable request.
• Code: R scripts of this study are available from the lead contact upon reasonable request.
• Other: No additional resources are available.

## Acknowledgments

The authors gratefully acknowledge the financial support from the 10.13039/501100018542Sichuan Natural Science Foundation (No. 2024NSFSC0691), the Project of Sichuan Traditional Chinese Medicine Administration (No. 2023MS071), and Sichuan Province “Tianfu Qingcheng Plan” Youth Science and Technology Talent Project (No. 1676).

## Author contributions

X.G. and Y.C. conducted the article and wrote the manuscript. Y.Z. and X.H. revised the manuscript. Y.S., L.W., Y.L.,Y.H., J.J., X. Zhang, S.P. and X. Zhou searched and collated the references. Y.L., J.Z. and G.F. conceived and designed the article.

## Declaration of interests

The authors declare no competing interest.

## STAR★Methods

### Key resources table

**Table:** 

REAGENT or RESOURCE	SOURCE	IDENTIFIER
Magnetic Soil and Stool DNA Kit	TianGen, China	DP712
USEARCH (v10.0)	Edgar RC	http://www.drive5.com/usearch/
Silva Database (v123)	ARB-SILVA	http://www.arb-silva.de/
Unite Database	UNITE Community	https://unite.ut.ee/
EasyAmplicon (v1.19)	Liu et al.^34^	

### Experimental model and study participant details

This study recruited 179 adults including 58 DMA subjects, 71 T2D subjects and 50 healthy controls. T2D participants were diagnosed by physicians according to the 1999 WHO criteria.^35^ Subsequently, participants diagnosed with T2D were assigned to the DMA subjects if they met at least one of the following criteria: diagnosis of coronary artery disease (CAD), cerebral infarction, or peripheral vascular ischemic disease by the attending medical specialist based on clinical symptoms, signs, identification of atherosclerotic plaques in peripheral blood vessels (such as bilateral carotid arteries, lower-extremity arteries, etc.), Ankle-Brachial Index (ABI) less than 0.9, or presence of increased intima-medial thickness (IMT) in the common carotid artery (CCA) (>1.1 mm) observed using color Doppler ultrasound examination.^36^^,^^37^^,^^38^^,^^39^ Participants were excluded if they had renal failure, kidney transplantation, gastrointestinal diseases, malignancy, infectious diseases, or other conditions known to affect gut microbes, or had received antibiotics, probiotics, or immunosuppressive medications in the previous 3 months. Clinical data are presented in Table 1. Ethical approval was obtained from the Medical Ethics Committee of the Affiliated Hospital of Chengdu University of Traditional Chinese Medicine (2020KL-060), and informed consent was obtained from all participants. All procedures followed were in accordance with the ethical standards of the responsible committee on human experimentation and with the Helsinki Declaration.

### Method details

#### DNA extraction from feces

Genomic DNA extraction was performed using Magnetic Soil and Stool DNA Kit (TianGen, China, Catalog #: DP712), following the manufacturer’s instructions. DNA concentration and purity were assessed using 1% agarose gel electrophoresis. Based on the concentration, DNA was diluted to 1 ng/μL using sterile water. The collected DNA was stored at −20°C until amplification by PCR.

#### Sequencing processing and filtering

The bioinformatic analyses of 16S amplicons were performed using EasyAmplicon (Version 1.19).^34^ Dereplication was achieved using VSEARCH (version 2.22.1),^40^ and the sequences were denoised into amplicon sequence variants (ASVs) using the implemented command in USEARCH (Version 10.0),^41^ resulting in the generation of an ASV Table. Taxonomic information for each representative 16S rRNA sequence was annotated using the Silva Database (http://www.arb-silva.de/) (Version 123) in conjunction with USEARCH (Version 10.0).^42^ Similarly, taxonomic information for each representative ITS sequence was annotated using the Unite Database (https://unite.ut.ee/) in conjunction with USEARCH (Version 10.0). The similarity threshold was set at 0.97, and the confidence threshold for Operational Taxonomic Unit (OTU) classification was set at 0.8.

#### PCR amplification and sequencing

Bacterial 16S ribosomal RNA (rRNA) gene sequences were also analyzed. The V4 region of the bacterial 16S rRNA genes was amplified by PCR using primers 515F and 806R. Fungal genomic sequences present in fecal samples were identified by amplification of the ITS region. The fungal diversity was identified using the ITS1 region primers (ITS5-1737F and ITS2-2043R). PCR reactions were carried out using 15 μL of Phusion High-Fidelity PCR Master Mix (New England Biolabs). The PCR amplification mixture contained about 10 ng of template DNA and 2 μM of forward and reverse primers. Thermal cycling consisted of initial denaturation at 98°C for 1 min, followed by 30 cycles of denaturation at 98°C for 10 s, annealing at 50°C for 30 s, and elongation at 72°C for 30 s and 72°C for 5 min. PCR products were purified using the Qiagen Gel Extraction Kit (Qiagen, Germany). Sequencing libraries were generated using the TruSeq DNA PCR-Free Sample Preparation Kit (Illumina, USA) following the manufacturer’s recommendations, and index codes were added. The library quality was assessed using the Qubit 2.0 Fluorometer (Thermo Scientific) and Agilent Bioanalyzer 2100 system. Quantified libraries were pooled and sequenced on Illumina platforms, according to effective library concentration and data amount required. The sequencing was performed on an Illumina NovaSeq 6000 platform, and 250 bp paired-end reads were generated.

### Quantification and statistical analysis

The abundance Tables of the gut bacteriome and mycobiome were imported into the R environment (version 4.3.1) for statistical analysis. Alpha diversity was computed using the vegan package in R. Statistical significance of differences in alpha diversity indices between three groups was assessed using the Kruskal-Wallis H test. Beta diversity analysis was performed using constrained principal coordinates analysis (CPCoA) based on Bray-Curtis distance, as implemented in the vegan package in R. Both alpha and beta diversity were visualized using the ggplot2 package in R. Taxonomic composition was depicted using ggplot2, presenting a stacked bar plot at various taxonomic levels.

Multivariate association with linear models (MaAsLin2) was employed to identify differential microbial taxa between groups, adjusting for confounding variables such as age, sex, BMI, SBP and DBP.^43^ Taxa with relative abundance exceeding 0.01% and prevalence surpassing 10% were exclusively chosen for MaAsLin2 analysis. For inter-group ASV comparisons, the Kruskal-Wallis H method was employed to compute the P-value and discern taxonomic features significantly varying among groups, with false discovery rate (FDR) calculated using the Benjamini-Hochberg method (*p* < 0.05). Moreover, the random forest (RF) algorithm was used to construct a classification model to explore the potential of gut microbiome in disease diagnosis and prediction. It was performed in R using the randomForest package. In each case, 70% of the samples were used for model training and the remaining 30% were used to test the performance of the model. Parameters were optimized using 5-fold cross validation. The receiver operating characteristic (ROC) curves of three classification models were plotted using the macro-average algorithm, respectively. All statistical tests and significance values are detailed in figure legends and Results.

### Additional resources

#### Clinical trial registry numbers

The clinical trial has been registered in the Chinese Clinical Trial Registry (No. ChiCTR2000040870) https://www.chictr.org.cn/hvshowproject.html?id=67111&v=1.0.

## Supplemental information

Document S1. Figures S1–S7 and Tables S2–S5 and S7–S13

Table S1. Read statistics for bacterial 16S rRNA sequencing data

Table S6. Read statistics for fungal genomic analysis sequencing data

## References

[1] Madonna R., Pieragostino D., Balistreri C.R., Rossi C., Geng Y.J., Del Boccio P., De Caterina R. (2018). Diabetic macroangiopathy: Pathogenetic insights and novel therapeutic approaches with focus on high glucose-mediated vascular damage. Vascul. Pharmacol..

[2] Einarson T.R., Acs A., Ludwig C., Panton U.H. (2018). Prevalence of cardiovascular disease in type 2 diabetes: a systematic literature review of scientific evidence from across the world in 2007-2017. Cardiovasc. Diabetol..

[3] Jahan H., Choudhary M.I. (2021). Gliclazide alters macrophages polarization state in diabetic atherosclerosis in vitro via blocking AGE-RAGE/TLR4-reactive oxygen species-activated NF-kβ nexus. Eur. J. Pharmacol..

[4] Osmenda G., Matusik P.T., Sliwa T., Czesnikiewicz-Guzik M., Skupien J., Malecki M.T., Siedlinski M. (2021). Nicotinamide adenine dinucleotide phosphate (NADPH) oxidase p22phox subunit polymorphisms, systemic oxidative stress, endothelial dysfunction, and atherosclerosis in type 2 diabetes mellitus. Pol. Arch. Intern. Med..

[5] Loftus M., Hassouneh S.A.D., Yooseph S. (2021). Bacterial associations in the healthy human gut microbiome across populations. Sci. Rep..

[6] Santus W., Devlin J.R., Behnsen J. (2021). Crossing kingdoms: How the mycobiota and fungal-bacterial interactions impact host health and disease. Infect. Immun..

[7] Lima S.F., Pires S., Rupert A., Oguntunmibi S., Jin W.B., Marderstein A., Funez-dePagnier G., Maldarelli G., Viladomiu M., Putzel G. (2024). The gut microbiome regulates the clinical efficacy of sulfasalazine therapy for IBD-associated spondyloarthritis. Cell Rep. Med..

[8] Zeng F., Su X., Liang X., Liao M., Zhong H., Xu J., Gou W., Zhang X., Shen L., Zheng J.S., Chen Y.M. (2024). Gut microbiome features and metabolites in non-alcoholic fatty liver disease among community-dwelling middle-aged and older adults. BMC Med..

[9] Avery E.G., Bartolomaeus H., Rauch A., Chen C.Y., N'Diaye G., Löber U., Bartolomaeus T.U.P., Fritsche-Guenther R., Rodrigues A.F., Yarritu A. (2023). Quantifying the impact of gut microbiota on inflammation and hypertensive organ damage. Cardiovasc. Res..

[10] Fan G., Cao F., Kuang T., Yi H., Zhao C., Wang L., Peng J., Zhuang Z., Xu T., Luo Y. (2023). Alterations in the gut virome are associated with type 2 diabetes and diabetic nephropathy. Gut Microbes.

[11] Collins S.L., Stine J.G., Bisanz J.E., Okafor C.D., Patterson A.D. (2023). Bile acids and the gut microbiota: metabolic interactions and impacts on disease. Nat. Rev. Microbiol..

[12] Du L., Li Q., Yi H., Kuang T., Tang Y., Fan G. (2022). Gut microbiota-derived metabolites as key actors in type 2 diabetes mellitus. Biomed. Pharmacother..

[13] Demir M., Lang S., Hartmann P., Duan Y., Martin A., Miyamoto Y., Bondareva M., Zhang X., Wang Y., Kasper P. (2022). The fecal mycobiome in non-alcoholic fatty liver disease. J. Hepatol..

[14] Gutierrez M.W., Mercer E.M., Moossavi S., Laforest-Lapointe I., Reyna M.E., Becker A.B., Simons E., Mandhane P.J., Turvey S.E., Moraes T.J. (2023). Maturational patterns of the infant gut mycobiome are associated with early-life body mass index. Cell Rep. Med..

[15] Wang L., Zhang K., Zeng Y., Luo Y., Peng J., Zhang J., Kuang T., Fan G. (2023). Gut mycobiome and metabolic diseases: The known, the unknown, and the future. Pharmacol. Res..

[16] Chen Z., Radjabzadeh D., Chen L., Kurilshikov A., Kavousi M., Ahmadizar F., Ikram M.A., Uitterlinden A.G., Zhernakova A., Fu J. (2021). Association of Insulin Resistance and Type 2 Diabetes With Gut Microbial Diversity: A Microbiome-Wide Analysis From Population Studies. JAMA Netw. Open.

[17] Das T., Jayasudha R., Chakravarthy S., Prashanthi G.S., Bhargava A., Tyagi M., Rani P.K., Pappuru R.R., Sharma S., Shivaji S. (2021). Alterations in the gut bacterial microbiome in people with type 2 diabetes mellitus and diabetic retinopathy. Sci. Rep..

[18] Jayasudha R., Das T., Kalyana Chakravarthy S., Sai Prashanthi G., Bhargava A., Tyagi M., Rani P.K., Pappuru R.R., Shivaji S. (2020). Gut mycobiomes are altered in people with type 2 Diabetes Mellitus and Diabetic Retinopathy. PLoS One.

[19] Aguirre de Cárcer D., Cuív P.O., Wang T., Kang S., Worthley D., Whitehall V., Gordon I., McSweeney C., Leggett B., Morrison M. (2011). Numerical ecology validates a biogeographical distribution and gender-based effect on mucosa-associated bacteria along the human colon. ISME J.W.

[20] Deb D., Das S., Adak A., Khan M.R. (2020). Traditional rice beer depletes butyric acid-producing gut bacteria Faecalibacterium and Roseburia along with fecal butyrate levels in the ethnic groups of Northeast India. 3 Biotech.

[21] Duncan S.H., Holtrop G., Lobley G.E., Calder A.G., Stewart C.S., Flint H.J. (2004). Contribution of acetate to butyrate formation by human faecal bacteria. Br. J. Nutr..

[22] Gózd-Barszczewska A., Kozioł-Montewka M., Barszczewski P., Młodzińska A., Humińska K. (2017). Gut microbiome as a biomarker of cardiometabolic disorders. Ann. Agric. Environ. Med..

[23] Martín R., Rios-Covian D., Huillet E., Auger S., Khazaal S., Bermúdez-Humarán L.G., Sokol H., Chatel J.M., Langella P. (2023). Faecalibacterium: A bacterial genus with promising human health applications. FEMS Microbiol. Rev..

[24] Bank N.C., Singh V., Rodriguez-Palacios A. (2022). Classification of Parabacteroides distasonis and other Bacteroidetes using O- antigen virulence gene: RfbA-Typing and hypothesis for pathogenic vs. probiotic strain differentiation. Gut Microbes.

[25] Wang K., Liao M., Zhou N., Bao L., Ma K., Zheng Z., Wang Y., Liu C., Wang W., Wang J. (2019). Parabacteroides distasonis Alleviates Obesity and Metabolic Dysfunctions via Production of Succinate and Secondary Bile Acids. Cell Rep..

[26] Li M., Wang S., Li Y., Zhao M., Kuang J., Liang D., Wang J., Wei M., Rajani C., Ma X. (2022). Gut microbiota-bile acid crosstalk contributes to the rebound weight gain after calorie restriction in mice. Nat. Commun..

[27] Helaly S.E., Thongbai B., Stadler M. (2018). Diversity of biologically active secondary metabolites from endophytic and saprotrophic fungi of the ascomycete order Xylariales. Nat. Prod. Rep..

[28] Martin F.M., Uroz S., Barker D.G. (2017). Ancestral alliances: Plant mutualistic symbioses with fungi and bacteria. Science.

[29] Fawagreh K., Gaber M.M., Elyan E. (2014). Random Forests: From Early Developments to Recent Advancements. Syst. Sci. Control Eng..

[30] Luan J., Zhang C., Xu B., Xue Y., Ren Y. (2020). The predictive performances of random forest models with limited sample size and different species traits. Fish. Res..

[31] Capitaine L., Genuer R., Thiébaut R. (2021). Random forests for high-dimensional longitudinal data. Stat. Methods Med. Res..

[32] Saxena A., Mathur N., Pathak P., Tiwari P., Mathur S.K. (2023). Machine Learning Model Based on Insulin Resistance Metagenes Underpins Genetic Basis of Type 2 Diabetes. Biomolecules.

[33] Lone I.M., Nun N.B., Ghnaim A., Schaefer A.S., Houri-Haddad Y., Iraqi F.A. (2023). High-fat diet and oral infection induced type 2 diabetes and obesity development under different genetic backgrounds. Animal Model. Exp. Med..

[34] Liu Y.X., Qin Y., Chen T., Lu M., Qian X., Guo X., Bai Y. (2021). A practical guide to amplicon and metagenomic analysis of microbiome data. Protein Cell.

[35] Gabir M.M., Hanson R.L., Dabelea D., Imperatore G., Roumain J., Bennett P.H., Knowler W.C. (2000). The 1997 American Diabetes Association and 1999 World Health Organization criteria for hyperglycemia in the diagnosis and prediction of diabetes. Diabetes Care.

[36] Li M. (2021). Guidelines and standards for comprehensive clinical diagnosis and interventional treatment for diabetic foot in China (Issue 7.0). J. Interv. Med..

[37] Chen T., Tu M., Huang L., Zheng Y. (2020). Association of Serum Adiponectin with Intima Media Thickness of Dorsalis Pedis Artery and Macroangiopathy in Type 2 Diabetes. J. Diabetes Res..

[38] Zhang S., Wu H. (2024). Research progress in clinical diagnosis of diabetic macrovascular diseas. Int. J. Lab. Med..

[39] Yin Y., Wang H., Zhou J., Song Z., Zhao Q. (2021). Clinical Study of Huangqi (Astragali Radix)-Danggui (Angelicae Sinensis Radix) on Intervention of 8-iso-PGF2 α in Serum of T2DM Patients with Macroangiopathy of Qi Deficiency and Blood Stasis Type. Chinese Arch. Tradit. Chinese Med..

[40] Rognes T., Flouri T., Nichols B., Quince C., Mahé F. (2016). VSEARCH: a versatile open source tool for metagenomics. PeerJ.

[41] Edgar R.C. (2010). Search and clustering orders of magnitude faster than BLAST. Bioinformatics.

[42] Quast C., Pruesse E., Yilmaz P., Gerken J., Schweer T., Yarza P., Peplies J., Glöckner F.O. (2013). The SILVA ribosomal RNA gene database project: improved data processing and web-based tools. Nucleic Acids Res..

[43] Mallick H., Rahnavard A., McIver L.J., Ma S., Zhang Y., Nguyen L.H., Tickle T.L., Weingart G., Ren B., Schwager E.H. (2021). Multivariable association discovery in population-scale meta-omics studies. PLoS Comput. Biol..

