# Supplementary material for: Disturbance of gut microbiota in diabetes related macroangiopathy: Evidence from the gut bacteriome and mycobiome
Source: iScience. 2025 Jun 9;28(7):112856. doi: 10.1016/j.isci.2025.112856 (PMC12268692; doi:10.1016/j.isci.2025.112856)
Supplement: Document S1. Figures S1–S7 and Tables S2–S5 and S7–S13 [file mmc1.pdf]

iScience, Volume *28*

Supplemental information

Disturbance of gut microbiota in diabetes related macroangiopathy: Evidence from the gut bacteriome and mycobiome

Xiaoling Gou, Yuqing Chen, Yi Zong, Xuemei Huang, Yihong Shen, Lijie Wang, Yifan Liu, Yuchi He, Jialong Jia, Xiyu Zhang, Sihan Peng, Xianhua Zhou, Ya Liu, Jing Zhang, and Gang Fan

Figure S1

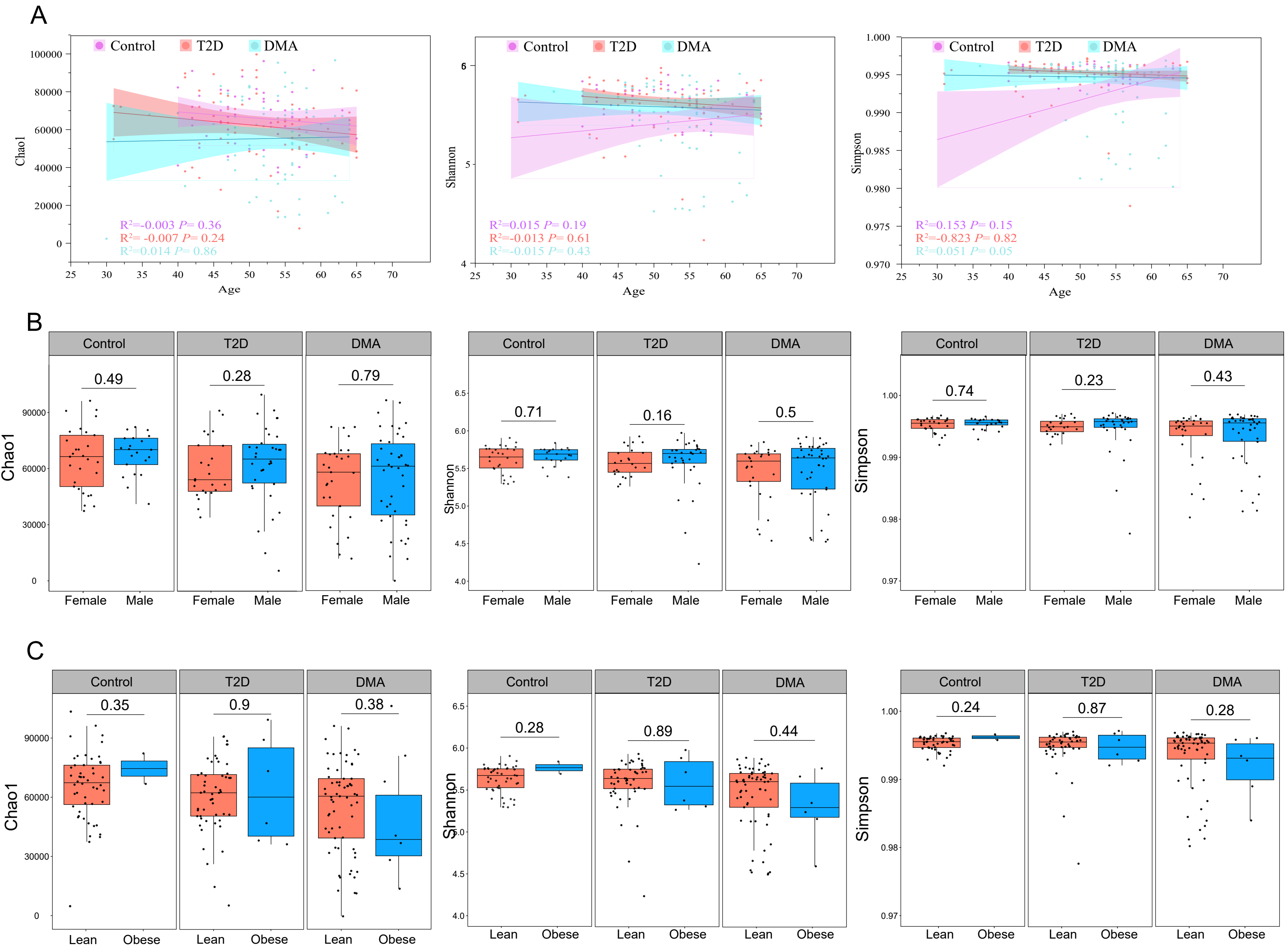

Figure S1—The association of age, sex, and obesity with gut bacterial alpha diversity in T2D, DMA and Control groups. (A) Chao1, Shannon and Simpson indices correlate with age. Statistical significance was determined by linear regression. Comparison of the 3 diversity indices between (B) males and females, and (C) obese and lean. Statistical significance was determined by Wilcoxon's rank um test. For the box plots, the boxes extend from the first to the third quartile (25th to 75th percentiles), with the center line indicating the median.

Figure S2

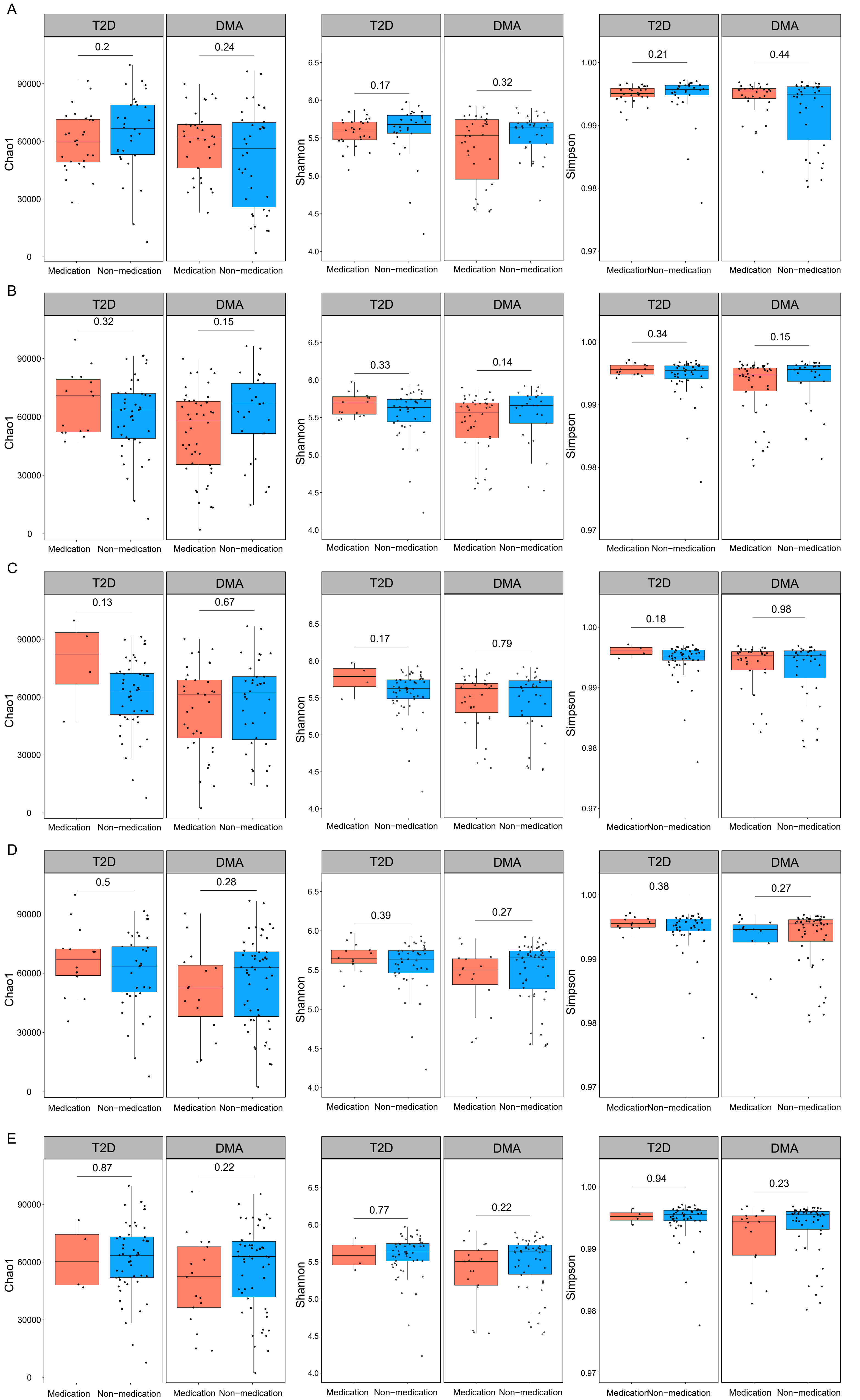

Figure S2—Effects of several commonly used drugs on bacterial diversity in patients with T2D and DMA, including (A) metformin, (B) statins, (C) non-steroidal anti-inflammatory drugs (NSAIDs), (D) sulfonylureas and (E) angiotensin Receptor Blockers (ARBS). No significant difference was observed in the 3 diversity indices between medication and non-medication. For the box plots, the boxes extend from the first to the third quartile (25th to 75th percentiles), with the center line indicating the median.

Figure S3

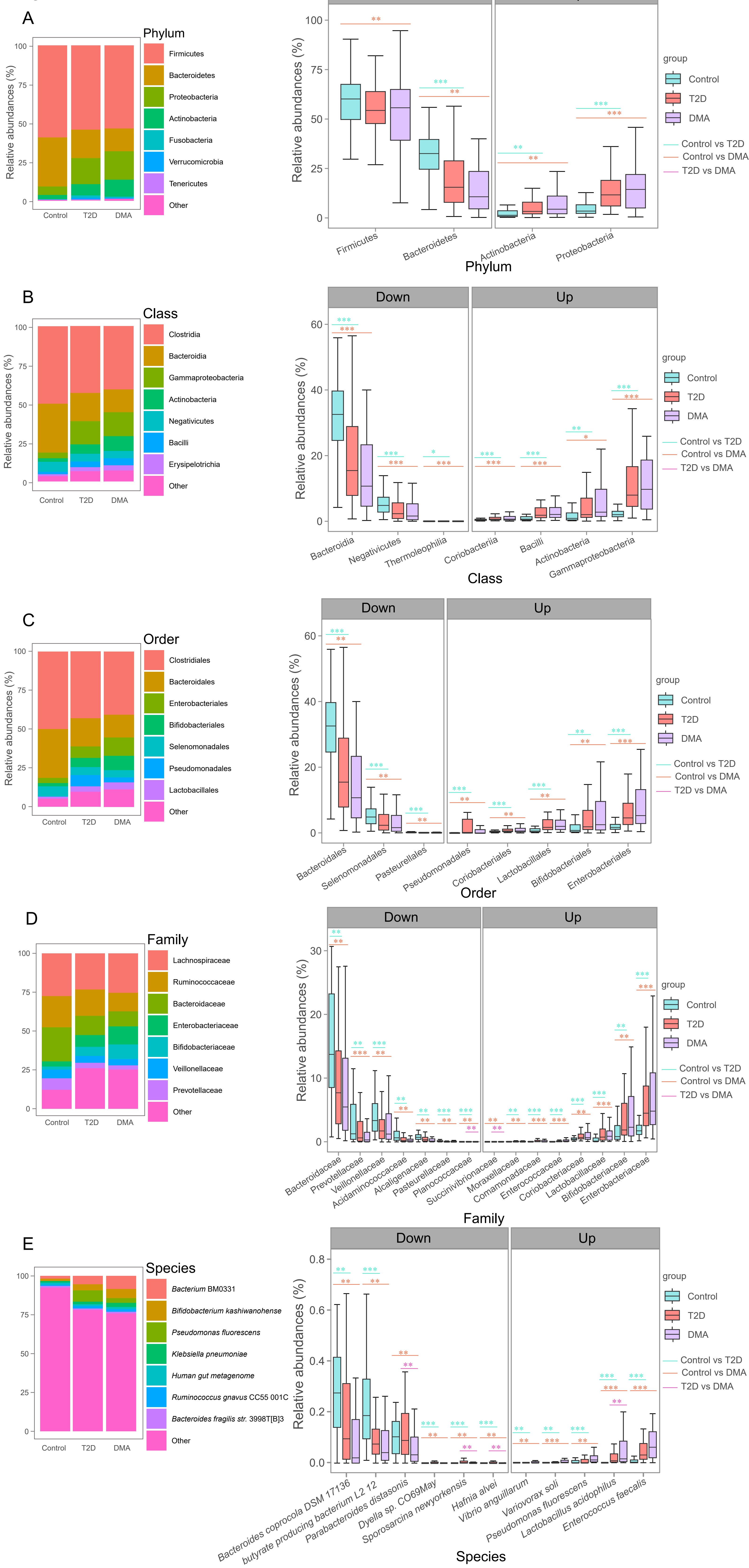

Figure S3—Alteration of gut bacteriome at (A) Phylum, (B) Class , (C) Order , (D) Family and (E) Species levels in DMA compared with T2D and controls. The taxonomic distribution of bacterial taxa is shown in the bar plot on the left. Differential bacterial taxa identified by MaAsLin2 after adjusting for confounders are shown in the box plots on the right.

Figure S4

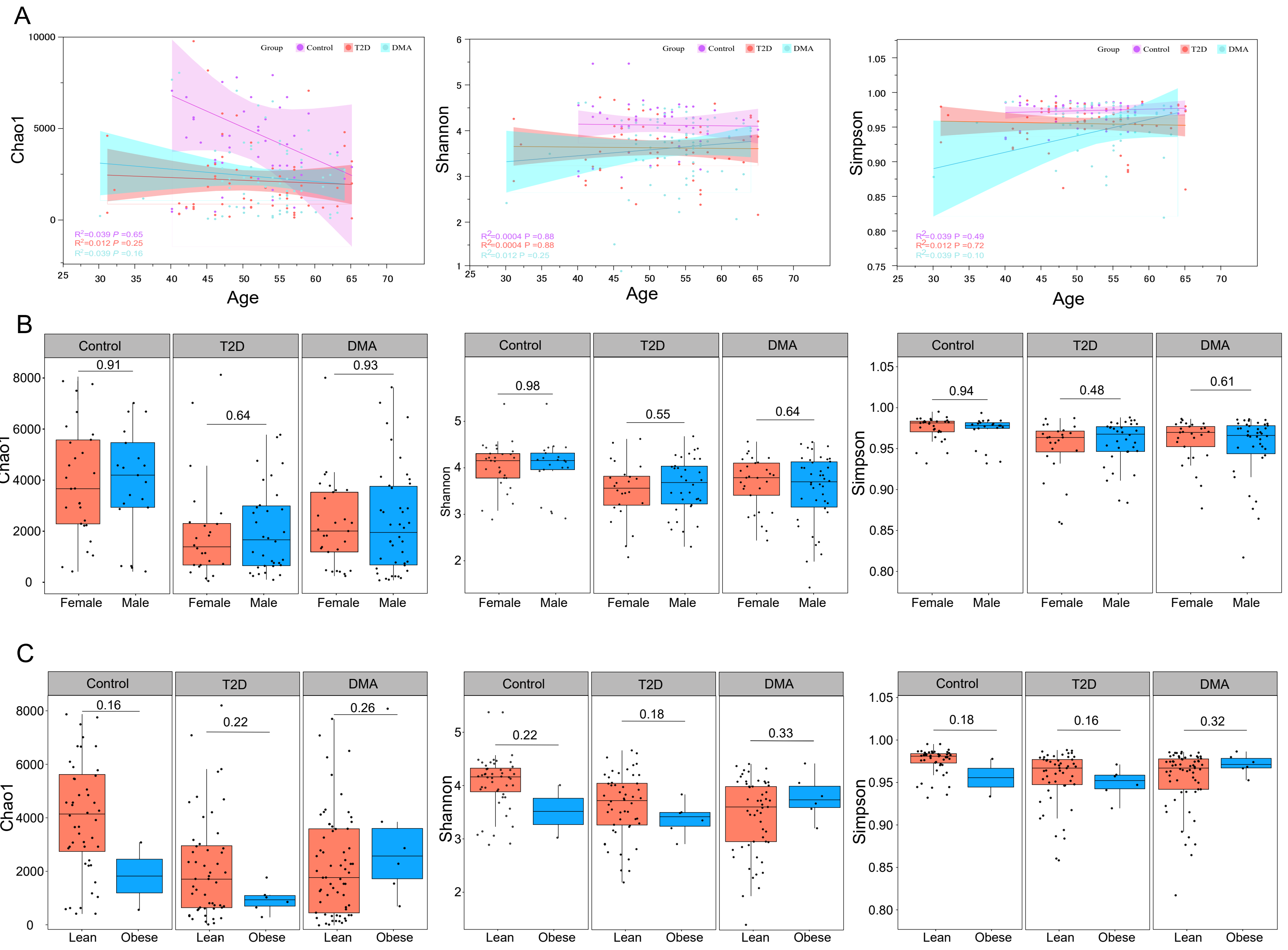

Figure S4—The association of age, sex, and obesity with gut fungal alpha diversity in T2D, DMA and Control groups. (A) Chao1, Shannon and Simpson indices correlate with age. Statistical significance was determined by linear regression. Comparison of the 3 diversity indices between (B) males and females, and (C) obese and lean. Statistical significance was determined by Wilcoxon's rank sum test. For the box plots, the boxes extend from the first to the third quartile (25th to 75th percentiles), with the center line indicating the median.

Figure S5

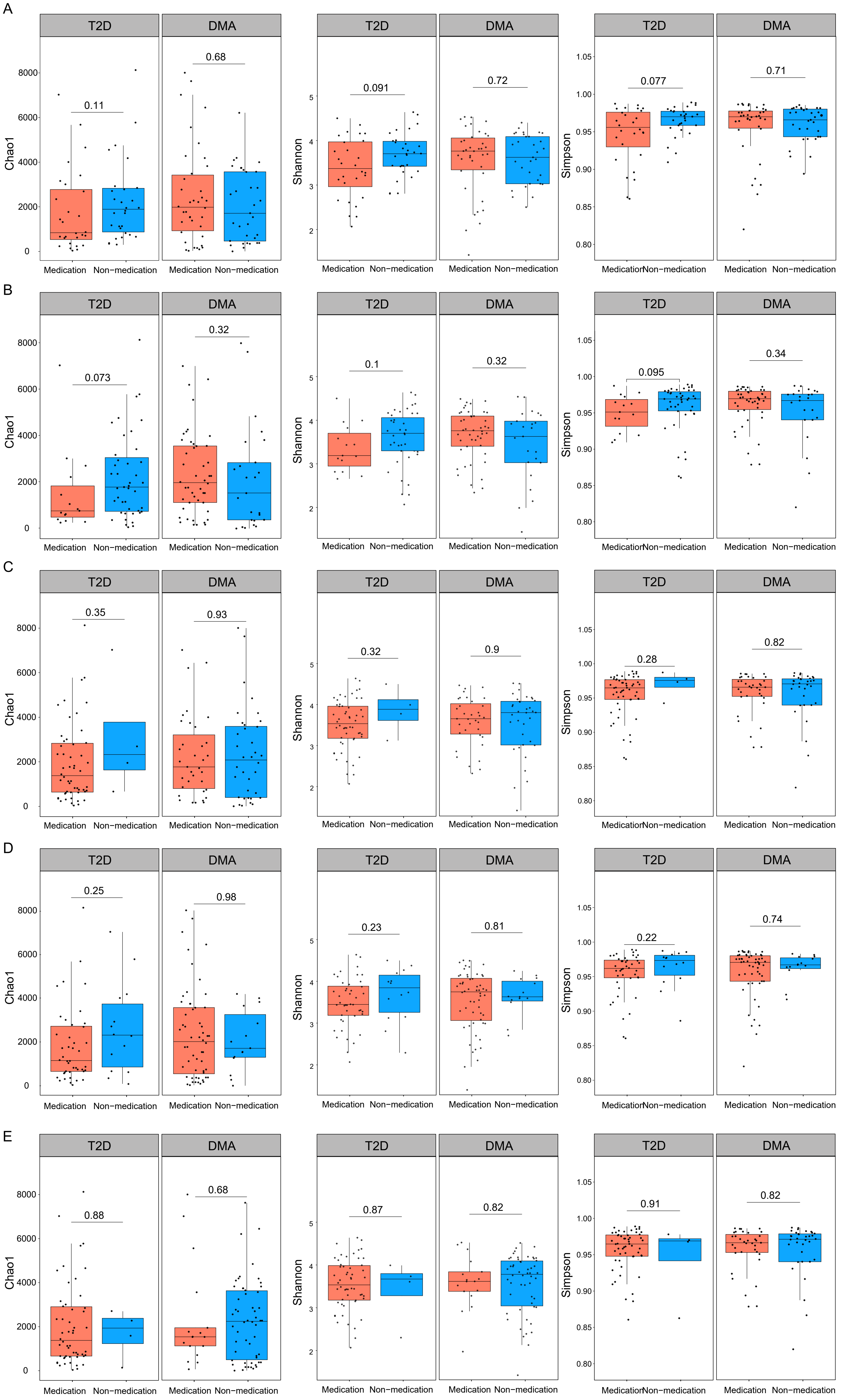

Figure S5—Effects of several commonly used drugs on bacterial diversity in patients with T2D and DMA, including (A) metformin, (B) statins, (C) non-steroidal anti-inflammatory drugs (NSAIDs), (D) sulfonylureas and (E) angiotensin Receptor Blockers (ARBS). No significant difference was observed in the 3 diversity indices between medication and non-medication. For the box plots, the boxes extend from the first to the third quartile (25th to 75th percentiles), with the center line indicating the median.

Figure S6

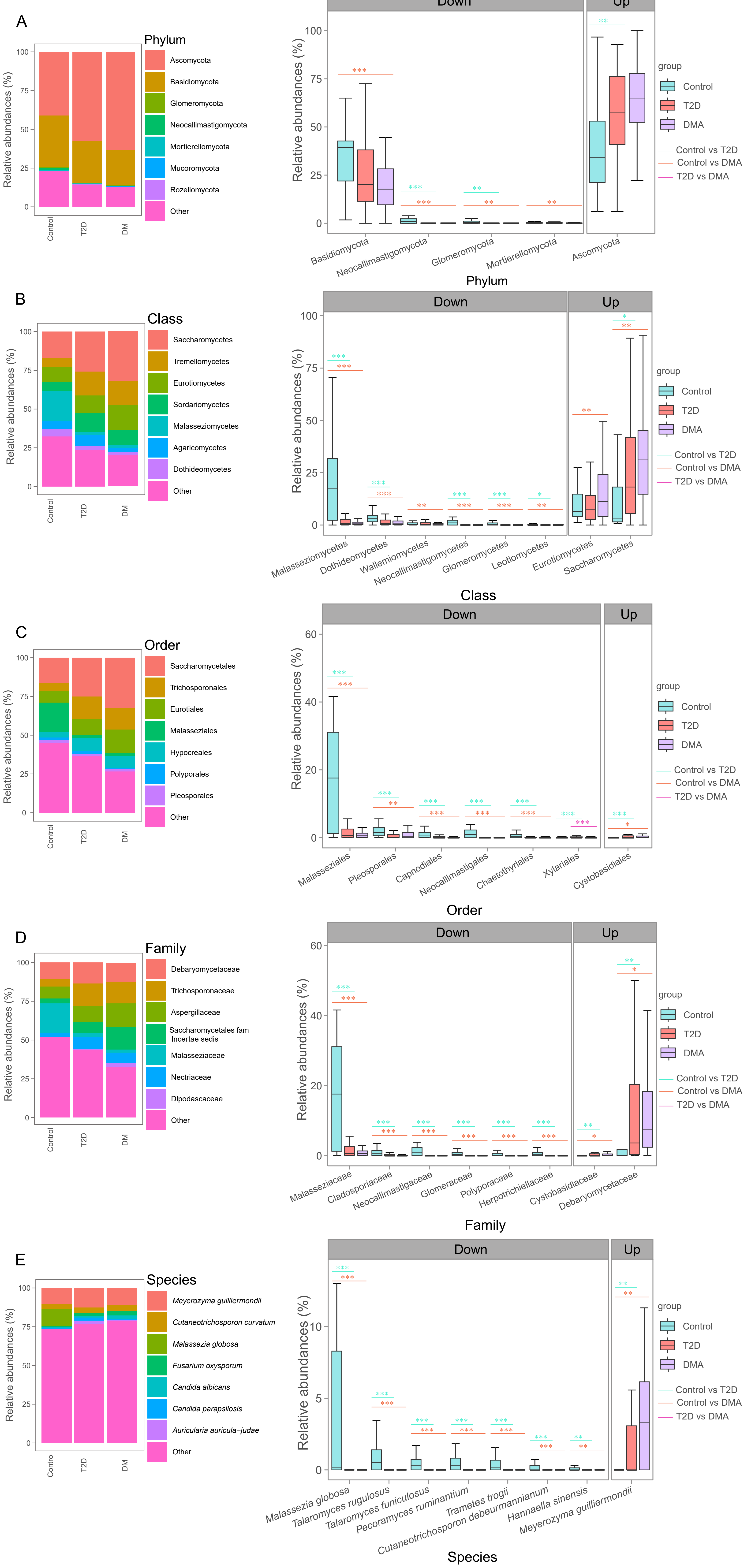

Figure S6—Alteration of gut mycobiome at (A) Phylum, (B) Class , (C) Order , (D) Family and (E) Species levels in DMA compared with T2D and controls. The taxonomic distribution of bacterial taxa is shown in the bar plot on the left. Differential bacterial taxa identified by MaAsLin2 after adjusting for confounders are shown in the box plots on the right.

Figure S7

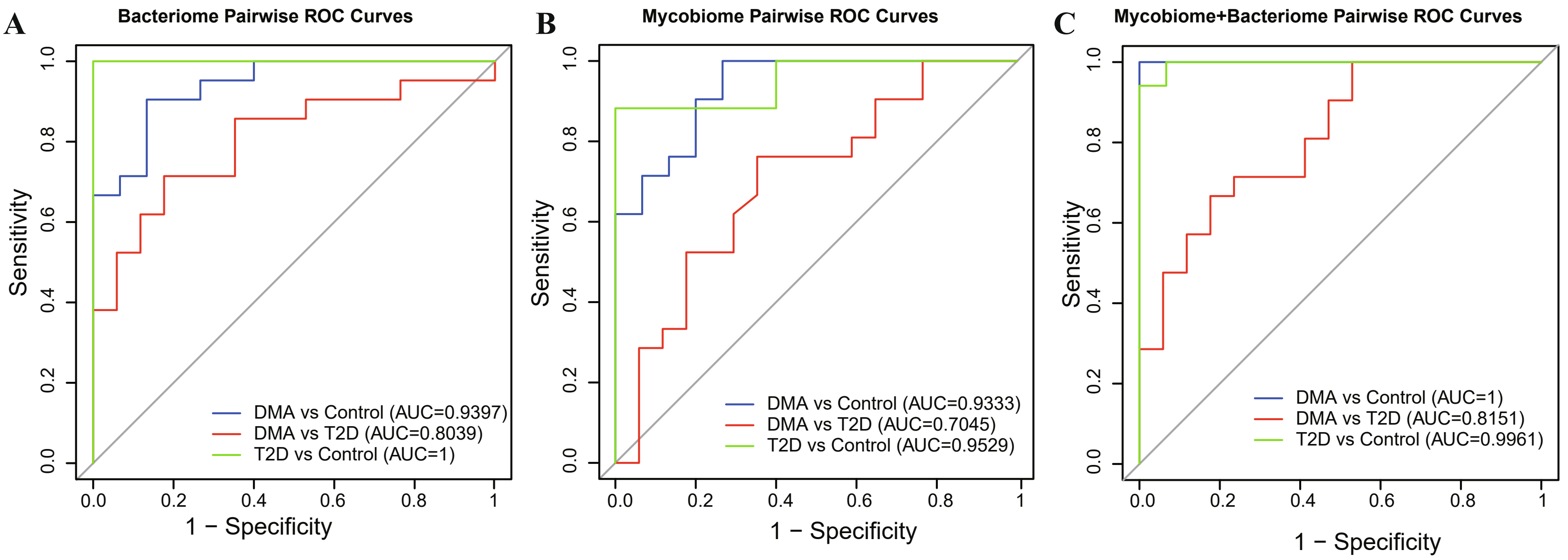

Figure S7—ROC curves of random forest models based on the microbiome (bacteriome, mycobiome, and their combination) for distinguishing between DMA, T2D and healthy controls. (A) Model based on the bacteriome; (B) model based on the mycobiome; (C) model based on the combined bacteriome and mycobiome. Each panel presents ROC curves and the corresponding AUC values for three pairwise comparisons: DMA vs. Control, DMA vs. T2D, and T2D vs. Control. ROC curves were generated based on predicted probabilities from the test set. Models were trained using 10-fold cross-validation and optimized via grid search for the parameter mtry.

Table S2 Differences in bacterial orders among healthy controls, T2D, and DMA revealed by MaAsLin2.

feature	Compare	metadata	value	coef	stderr	N	N.not.0	pval	qval
Selenomonadales	Control vs. DMA	Group	DMA	-1.745334775	0.532480403	121	121	0.001384182	0.038649998
Pasteurellales	Control vs. DMA	Group	DMA	-1.717671773	0.559579381	121	119	0.002674597	0.053491935
Bacteroidales	Control vs. DMA	Group	DMA	-1.384150793	0.414702354	121	121	0.001138635	0.038649998
Clostridiales	Control vs. DMA	Group	DMA	-0.454295679	0.210654052	121	121	0.033119225	0.231834574
Coriobacteriales	Control vs. DMA	Group	DMA	0.838903476	0.334009707	121	121	0.013405884	0.156401984
Micrococcales	Control vs. DMA	Group	DMA	1.096092499	0.502727331	121	109	0.031273047	0.231834574
Lactobacillales	Control vs. DMA	Group	DMA	1.232346246	0.382486196	121	121	0.001656428	0.038649998
Bifidobacteriales	Control vs. DMA	Group	DMA	1.292839171	0.556005071	121	121	0.021813394	0.21382349
Enterobacteriales	Control vs. DMA	Group	DMA	1.491997623	0.411094194	121	121	0.000425409	0.028082505
Rhizobiales	Control vs. DMA	Group	DMA	1.744797592	0.753937888	121	75	0.022429724	0.21382349
Pseudomonadales	Control vs. DMA	Group	DMA	2.325016037	0.890567624	121	114	0.010238662	0.130310242
Xanthomonadales	Control vs. DMA	Group	DM	3.117439328	0.612090499	121	74	1.39E-06	0.000194926
Pasteurellales	Control vs. T2D	Group	T2D	-1.545233556	0.43751385	108	108	0.000621243	0.009663781
Selenomonadales	Control vs. T2D	Group	T2D	-1.537969883	0.390735585	108	108	0.000151495	0.003534889
Bacteroidales	Control vs. T2D	Group	T2D	-1.108570124	0.299093	108	108	0.000341889	0.005983063
Bacillales	Control vs. T2D	Group	T2D	0.874223272	0.382743368	108	108	0.024437864	0.190072274
Bifidobacteriales	Control vs. T2D	Group	T2D	0.928408324	0.410755223	108	108	0.025930872	0.191069586
Coriobacteriales	Control vs. T2D	Group	T2D	0.997024346	0.267953061	108	108	0.000325123	0.005983063
Corynebacteriales	Control vs. T2D	Group	T2D	1.310889087	0.412914065	108	60	0.001983388	0.02524312
Lactobacillales	Control vs. T2D	Group	T2D	1.390432144	0.321346319	108	108	3.54E-05	0.001237299
Enterobacteriales	Control vs. T2D	Group	T2D	1.484644756	0.306645945	108	108	4.60E-06	0.000214515
Rhizobiales	Control vs. T2D	Group	T2D	1.614845491	0.61446435	108	64	0.009913459	0.092525621
Micrococcales	Control vs. T2D	Group	T2D	1.687832687	0.396292702	108	97	4.58E-05	0.001282075
Xanthomonadales	Control vs. T2D	Group	T2D	2.871711987	0.495298472	108	64	7.56E-08	5.29E-06
Pseudomonadales	Control vs. T2D	Group	T2D	4.72605111	0.797956694	108	103	4.31E-08	5.29E-06

Table S3 Differences in bacterial families among healthy controls, T2D, and DMA revealed by MaAsLin2.

feature	Compare	metadata	value	coef	stderr	N	N.not.0	pval	qval
Prevotellaceae	Control vs. DMA	Group	DMA	-2.945109129	0.805225511	121	120	0.000385674	0.018292575
Pasteurellaceae	Control vs. DMA	Group	DMA	-1.717671773	0.559579381	121	119	0.002674597	0.050003331
Veillonellaceae	Control vs. DMA	Group	DMA	-1.64666076	0.564137071	121	120	0.004226835	0.065968571
Alcaligenaceae	Control vs. DMA	Group	DMA	-1.64319952	0.52697217	121	121	0.002299501	0.046861652
Acidaminococcaceae	Control vs. DMA	Group	DMA	-1.545065405	0.569111164	121	120	0.007652066	0.096776134
Bacteroidales S24.7 group	Control vs. DMA	Group	DMA	-1.480787668	0.788881215	121	109	0.063042154	0.24654732
Leuconostocaceae	Control vs. DMA	Group	DMA	-1.201706782	0.562463267	121	99	0.034757639	0.189187151
Gaiellaceae	Control vs. DMA	Group	DMA	-1.197352894	0.419673351	121	34	0.005136192	0.071243948
CFT112H7	Control vs. DMA	Group	DMA	-1.163299068	0.57970754	121	40	0.047127669	0.217902124
Gemmatimonadaceae	Control vs. DMA	Group	DMA	-1.142638702	0.551574868	121	60	0.040540024	0.207526311
Bacteroidaceae	Control vs. DMA	Group	DMA	-1.093768907	0.422203215	121	121	0.010820115	0.11631624
X0319.6M6	Control vs. DMA	Group	DMA	-0.985685349	0.379729966	121	31	0.010668841	0.11631624
Rikenellaceae	Control vs. DMA	Group	DMA	-0.951591607	0.494870363	121	120	0.056964137	0.240142932
Ruminococcaceae	Control vs. DMA	Group	DMA	-0.809388792	0.268285326	121	121	0.003144504	0.054085465
Acidimicrobiaceae	Control vs. DMA	Group	DMA	-0.539751844	0.248449443	121	15	0.03187144	0.18166614
Family Incertae Sedis	Control vs. DMA	Group	DMA	0.545959177	0.237858881	121	15	0.023528552	0.160591703
Family XII	Control vs. DMA	Group	DMA	0.590745707	0.311020196	121	14	0.060019549	0.246332675
Planococcaceae	Control vs. DMA	Group	DMA	0.686865342	0.269024494	121	24	0.011983949	0.123648608
Solimonadaceae	Control vs. DMA	Group	DMA	0.767204731	0.247102198	121	15	0.002397573	0.046861652
Actinomycetaceae	Control vs. DMA	Group	DMA	0.781632404	0.396930341	121	118	0.051335991	0.232362908
Coriobacteriaceae	Control vs. DMA	Group	DMA	0.838903476	0.334009707	121	121	0.013405884	0.125070653
Vibrionaceae	Control vs. DMA	Group	DMA	0.906236378	0.464258879	121	39	0.053368338	0.232882523
Rhodobacteraceae	Control vs. DMA	Group	DMA	0.920181257	0.493867285	121	33	0.064983221	0.249489151
OM1 clade	Control vs. DMA	Group	DMA	0.948793206	0.428860018	121	28	0.028917393	0.17590407
Oxalobacteraceae	Control vs. DMA	Group	DMA	1.009256683	0.428566679	121	89	0.020219415	0.155256224
Micrococcaceae	Control vs. DMA	Group	DMA	1.096092499	0.502727331	121	109	0.031273047	0.18166614
Holophagaceae	Control vs. DMA	Group	DMA	1.141131155	0.332085039	121	13	0.000821565	0.024759906
Haliangiaceae	Control vs. DMA	Group	DMA	1.235496978	0.394801931	121	24	0.002220166	0.046861652
Moraxellaceae	Control vs. DMA	Group	DMA	1.256602513	0.641685731	121	104	0.052618322	0.232882523
Bifidobacteriaceae	Control vs. DMA	Group	DMA	1.292839171	0.556005071	121	121	0.021813394	0.158978972
Succinivibrionaceae	Control vs. DMA	Group	DMA	1.295764837	0.532323416	121	23	0.016464064	0.138814659
Enterobacteriaceae	Control vs. DMA	Group	DMA	1.491997623	0.411094194	121	121	0.000425409	0.018292575
Brucellaceae	Control vs. DMA	Group	DMA	1.571870575	0.411107123	121	35	0.000214135	0.013759313
Flavobacteriaceae	Control vs. DMA	Group	DMA	1.687409987	0.493210226	121	19	0.000863718	0.024759906
Aeromonadaceae	Control vs. DMA	Group	DMA	1.690436788	0.5602739	121	34	0.003141951	0.054085465
Nitrospiraceae	Control vs. DMA	Group	DMA	1.84977993	0.485381054	121	31	0.000223989	0.013759313
Eubacteriaceae	Control vs. DMA	Group	DMA	2.362944122	0.464984959	121	65	1.46E-06	0.000209539
Lactobacillaceae	Control vs. DMA	Group	DMA	2.463323263	0.555779211	121	121	2.14E-05	0.001842369
Pseudomonadaceae	Control vs. DMA	Group	DMA	2.744918188	0.942141973	121	104	0.004295628	0.065968571
Comamonadaceae	Control vs. DMA	Group	DMA	2.956768913	0.60834392	121	116	3.74E-06	0.000402135
Xanthomonadaceae	Control vs. DMA	Group	DMA	3.036610179	0.509042278	121	64	2.76E-08	1.19E-05
Enterococcaceae	Control vs. DMA	Group	DMA	3.668166525	0.684121778	121	106	4.31E-07	9.26E-05
Prevotellaceae	Control vs. T2D	Group	T2D	-2.001111785	0.631630628	108	108	0.002024528	0.035209187
Pasteurellaceae	Control vs. T2D	Group	T2D	-1.545233556	0.43751385	108	108	0.000621243	0.013805401
Acidaminococcaceae	Control vs. T2D	Group	T2D	-1.504026572	0.466529229	108	108	0.00169905	0.030891817
Veillonellaceae	Control vs. T2D	Group	T2D	-1.459905049	0.418894942	108	107	0.000726531	0.015295386
Gaiellaceae	Control vs. T2D	Group	T2D	-1.153727219	0.347150213	108	28	0.001235671	0.023536588
X0319.6M6	Control vs. T2D	Group	T2D	-1.085648153	0.303077557	108	23	0.000524187	0.012333802
Alcaligenaceae	Control vs. T2D	Group	T2D	-0.98915077	0.430257024	108	108	0.023545245	0.204094735
Bacteroidaceae	Control vs. T2D	Group	T2D	-0.948329836	0.320247034	108	108	0.003811545	0.058639156
Clostridiales vadinBB60 group	Control vs. T2D	Group	T2D	-0.934621855	0.425017717	108	30	0.030135574	0.236357442
Acidimicrobiaceae	Control vs. T2D	Group	T2D	-0.573065623	0.231498099	108	13	0.014953376	0.161658117
Family_XI	Control vs. T2D	Group	T2D	0.905075294	0.383726669	108	99	0.020247506	0.19283339
Rhodobacteraceae	Control vs. T2D	Group	T2D	0.919141909	0.385829349	108	25	0.019060293	0.190602932
Bifidobacteriaceae	Control vs. T2D	Group	T2D	0.928408324	0.410755223	108	108	0.025930872	0.216090603
Mycoplasmataceae	Control vs. T2D	Group	T2D	0.986193449	0.261120828	108	14	0.000267357	0.008226354
Helicobacteraceae	Control vs. T2D	Group	T2D	0.989890692	0.428785988	108	11	0.022983931	0.204094735
Coriobacteriaceae	Control vs. T2D	Group	T2D	0.997024346	0.267953061	108	108	0.000325123	0.009289219
Carnobacteriaceae	Control vs. T2D	Group	T2D	1.055236877	0.396120279	108	103	0.008978066	0.115846013
Corynebacteriaceae	Control vs. T2D	Group	T2D	1.320625107	0.362146475	108	39	0.000420362	0.011209656
Vibrionaceae	Control vs. T2D	Group	T2D	1.368534642	0.361473131	108	29	0.000258816	0.008226354
Planococcaceae	Control vs. T2D	Group	T2D	1.387393638	0.270801436	108	34	1.43E-06	9.51E-05
Enterobacteriaceae	Control vs. T2D	Group	T2D	1.484644756	0.306645945	108	108	4.60E-06	0.000229838
Eubacteriaceae	Control vs. T2D	Group	T2D	1.581346842	0.383539367	108	58	7.63E-05	0.002775784
Aeromonadaceae	Control vs. T2D	Group	T2D	1.585884351	0.340487371	108	27	9.67E-06	0.00042992
Moraxellaceae	Control vs. T2D	Group	T2D	1.611195362	0.514477496	108	94	0.002268064	0.037801074
Micrococcaceae	Control vs. T2D	Group	T2D	1.687832687	0.396292702	108	97	4.58E-05	0.001831536
Brucellaceae	Control vs. T2D	Group	T2D	1.970518486	0.33699781	108	38	6.05E-08	1.21E-05
Lactobacillaceae	Control vs. T2D	Group	T2D	2.376772111	0.480192802	108	108	2.95E-06	0.000168375
Comamonadaceae	Control vs. T2D	Group	T2D	2.682844718	0.515546934	108	100	1.01E-06	8.11E-05
Xanthomonadaceae	Control vs. T2D	Group	T2D	2.977986215	0.393459488	108	56	1.73E-11	6.94E-09
Enterococcaceae	Control vs. T2D	Group	T2D	3.021732366	0.570752113	108	92	6.89E-07	6.89E-05
Pseudomonadaceae	Control vs. T2D	Group	T2D	4.939384931	0.930700458	108	90	6.52E-07	6.89E-05
Succinivibrionaceae	DMA vs. T2D	Group	DMA	1.195115003	0.366308441	129	20	0.001429188	0.200564054
Planococcaceae	DMA vs. T2D	Group	DMA	-0.869184037	0.285896058	129	54	0.002889227	0.200564054

Table S4 Differences in bacterial genera among healthy controls, T2D, and DMA revealed by MaAsLin2.

feature	Compare	metadata	value	coef	stderr	N	N.not.0	pval	qval
Sarcina	Control vs. DMA	Group	DMA	4.800132149	0.819460727	121	70	4.55E-08	4.73E-05
Enterococcus	Control vs. DMA	Group	DMA	3.668166525	0.684121778	121	106	4.31E-07	0.000223933
Allobaculum	Control vs. DMA	Group	DMA	3.115142237	0.797269262	121	57	0.000158081	0.009670831
Pseudomonas	Control vs. DMA	Group	DMA	2.744918188	0.942141973	121	104	0.004295628	0.054521806
Lactobacillus	Control vs. DMA	Group	DMA	2.466562664	0.555722116	121	121	2.09E-05	0.002716496
Eubacterium	Control vs. DMA	Group	DMA	2.362944122	0.464984959	121	65	1.46E-06	0.000506791
Alcaligenes	Control vs. DMA	Group	DMA	1.89893681	0.544599359	121	19	0.000693085	0.020022447
Ralstonia	Control vs. DMA	Group	DMA	1.845482198	0.654638163	121	81	0.005672428	0.065548054
Lampropedia	Control vs. DMA	Group	DMA	1.766102853	0.477263853	121	45	0.000331743	0.012989726
Stenotrophomonas	Control vs. DMA	Group	DMA	1.726036829	0.473726373	121	43	0.000404916	0.013360796
Aeromonas	Control vs. DMA	Group	DMA	1.690436788	0.5602739	121	34	0.003141951	0.046022951
Eisenbergiella	Control vs. DMA	Group	DMA	1.624875777	0.462859139	121	72	0.000639702	0.0190083
Nitrospira	Control vs. DMA	Group	DMA	1.522469706	0.425232522	121	30	0.000503939	0.015881704
Variovorax	Control vs. DMA	Group	DMA	1.510825825	0.403561165	121	50	0.00028473	0.012989726
Pseudochrobactrum	Control vs. DMA	Group	DMA	1.50113681	0.388506997	121	35	0.000185106	0.010689427
Escherichia.Shigella	Control vs. DMA	Group	DMA	1.495999163	0.451299033	121	121	0.001226865	0.026039593
Klebsiella	Control vs. DMA	Group	DMA	1.435160724	0.525165252	121	121	0.007272583	0.077497077
Comamonas	Control vs. DMA	Group	DMA	1.379709363	0.671257755	121	71	0.042104199	0.212617177
Succinivibrio	Control vs. DMA	Group	DMA	1.295764837	0.532323416	121	23	0.016464064	0.126834272
Bifidobacterium	Control vs. DMA	Group	DMA	1.292839171	0.556005071	121	121	0.021813394	0.150400318
Acinetobacter	Control vs. DMA	Group	DMA	1.28646138	0.641890431	121	104	0.047401332	0.229290163
Pelomonas	Control vs. DMA	Group	DMA	1.277433117	0.34220044	121	34	0.000295771	0.012989726
Haliangium	Control vs. DMA	Group	DMA	1.235496978	0.394801931	121	24	0.002220166	0.037852017
GKS98 freshwater group	Control vs. DMA	Group	DMA	1.224567795	0.35776266	121	13	0.000859206	0.021794502
Cloacibacillus	Control vs. DMA	Group	DMA	1.192358406	0.322624558	121	25	0.000337233	0.012989726
Geothrix	Control vs. DMA	Group	DMA	1.141131155	0.332085039	121	13	0.000821565	0.021360694
X.Eubacterium. nodatum group	Control vs. DMA	Group	DMA	1.049638234	0.350224498	121	64	0.003340548	0.047314664
Delftia	Control vs. DMA	Group	DMA	1.044250048	0.41089055	121	52	0.012371233	0.104602292
Anaeroglobus	Control vs. DMA	Group	DMA	0.906728006	0.37205755	121	34	0.016339398	0.126813236
Vibrio	Control vs. DMA	Group	DMA	0.87842038	0.410952901	121	38	0.034673315	0.189181149
Morganella	Control vs. DMA	Group	DMA	0.821488218	0.338559789	121	22	0.016801855	0.127849582
Actinomyces	Control vs. DMA	Group	DMA	0.781632404	0.396930341	121	118	0.051335991	0.24451346
Hafnia	Control vs. DMA	Group	DMA	0.763913715	0.22748267	121	14	0.001064762	0.02407289
Lactonifactor	Control vs. DMA	Group	DMA	0.746762756	0.347324342	121	51	0.033646046	0.189181149
Atopobium	Control vs. DMA	Group	DMA	0.739560371	0.338189374	121	52	0.030780213	0.181883078
Olsenella	Control vs. DMA	Group	DMA	0.733277351	0.256132005	121	14	0.004989933	0.059649768
Phenylobacterium	Control vs. DMA	Group	DMA	0.71144747	0.324427084	121	28	0.030325152	0.180218049
Parvimonas	Control vs. DMA	Group	DMA	0.690476537	0.334885643	121	35	0.041477753	0.212617177
Sporosarcina	Control vs. DMA	Group	DMA	0.686865342	0.269024494	121	24	0.011983949	0.103002538
Mogibacterium	Control vs. DMA	Group	DMA	0.661313822	0.282529386	121	48	0.020971095	0.147754637
Acidovorax	Control vs. DMA	Group	DMA	0.557917754	0.200135171	121	25	0.00621276	0.070231199
Marinicella	Control vs. DMA	Group	DMA	0.545959177	0.237858881	121	15	0.023528552	0.151925648
Luteibacter	Control vs. DMA	Group	DMA	0.429157255	0.143315863	121	15	0.00336662	0.047314664
CL500.29 marine group	Control vs. DMA	Group	DMA	-0.539751844	0.248449443	121	15	0.03187144	0.185515397
Parapusillimonas	Control vs. DMA	Group	DMA	-0.653234581	0.286010485	121	22	0.024209758	0.153525293
Aggregatibacter	Control vs. DMA	Group	DMA	-0.676217989	0.293970913	121	36	0.02323502	0.151925648
Oscillospira	Control vs. DMA	Group	DMA	-0.681686049	0.281048015	121	40	0.016841724	0.127849582
Howardella	Control vs. DMA	Group	DMA	-0.805998923	0.273953674	121	32	0.003943445	0.051913704
Roseburia	Control vs. DMA	Group	DMA	-0.882518281	0.328655358	121	121	0.008319844	0.083198442
Lachnospiraceae ND3007 group	Control vs. DMA	Group	DMA	-0.964333139	0.446567304	121	116	0.032893151	0.187040268
Alistipes	Control vs. DMA	Group	DMA	-0.996240493	0.4924462	121	120	0.045387886	0.224778104
Prevotellaceae UCG.001	Control vs. DMA	Group	DMA	-1.046264395	0.323369998	121	21	0.001586001	0.030843944
Family XIII UCG.001	Control vs. DMA	Group	DMA	-1.0668522	0.418885803	121	79	0.01219007	0.103915347
Bacteroides	Control vs. DMA	Group	DMA	-1.093768907	0.422203215	121	121	0.010820115	0.100038961
Erysipelotrichaceae UCG.003	Control vs. DMA	Group	DMA	-1.115517142	0.526478968	121	120	0.036257279	0.194197614
Parabacteroides	Control vs. DMA	Group	DMA	-1.158736105	0.509362141	121	121	0.024766725	0.155641526
Pseudobutyrivibrio	Control vs. DMA	Group	DMA	-1.183990252	0.570690192	121	108	0.040248949	0.20879382
Allisonella	Control vs. DMA	Group	DMA	-1.193358751	0.518613573	121	92	0.023190114	0.151925648
Gaiella	Control vs. DMA	Group	DMA	-1.197352894	0.419673351	121	34	0.005136192	0.060700446
X.Eubacterium. ventriosum group	Control vs. DMA	Group	DMA	-1.210310978	0.461645027	121	119	0.009931762	0.093900297
Bilophila	Control vs. DMA	Group	DMA	-1.216695841	0.524426486	121	119	0.022102117	0.151225012
Ruminococcaceae UCG.002	Control vs. DMA	Group	DMA	-1.249731902	0.583926857	121	116	0.034449649	0.189181149
Ruminococcus 1	Control vs. DMA	Group	DMA	-1.470590495	0.476812424	121	119	0.002556103	0.040897643
X.Eubacterium. ruminantium group	Control vs. DMA	Group	DMA	-1.499324428	0.606965573	121	115	0.014971523	0.118204099
Phascolarctobacterium	Control vs. DMA	Group	DMA	-1.50274019	0.581811284	121	120	0.011052446	0.100038961
Ruminococcaceae NK4A214 group	Control vs. DMA	Group	DMA	-1.521713026	0.526480454	121	120	0.004601287	0.056872483
Lachnospiraceae UCG.005	Control vs. DMA	Group	DMA	-1.546145586	0.575170102	121	88	0.008251908	0.083198442
Prevotella 7	Control vs. DMA	Group	DMA	-1.562126309	0.66510536	121	58	0.020546668	0.147369203
Ruminiclostridium	Control vs. DMA	Group	DMA	-1.56432614	0.429666347	121	119	0.000408794	0.013360796
Incertae Sedis	Control vs. DMA	Group	DMA	-1.634142519	0.482851914	121	120	0.000976435	0.022579781
Haemophilus	Control vs. DMA	Group	DMA	-1.694474999	0.572247849	121	118	0.003724621	0.050306568
Weissella	Control vs. DMA	Group	DMA	-1.747729879	0.572296579	121	86	0.00280725	0.043064534
Dialister	Control vs. DMA	Group	DMA	-1.748328868	0.581193136	121	120	0.003229307	0.046645551
Lachnospiraceae UCG.001	Control vs. DMA	Group	DMA	-1.751533121	0.473098708	121	116	0.000329669	0.012989726
Burkholderia	Control vs. DMA	Group	DMA	-1.77721844	0.663700543	121	80	0.008496963	0.084160397
Butyricimonas	Control vs. DMA	Group	DMA	-1.817124383	0.536775194	121	94	0.000973511	0.022579781

Castellaniella	Control vs. DMA	Group	DMA	-1.861639293	0.423224677	121	43	2.45E-05	0.002825761
Holdemanella	Control vs. DMA	Group	DMA	-1.954585719	0.700927858	121	111	0.00619727	0.070231199
Barnesiella	Control vs. DMA	Group	DMA	-1.978075703	0.575550657	121	108	0.000819977	0.021360694
Lachnospiraceae UCG.010	Control vs. DMA	Group	DMA	-2.031556937	0.416188595	121	77	3.43E-06	0.000890689
Prevotellaceae Ga6A1 group	Control vs. DMA	Group	DMA	-2.055484131	0.473317424	121	26	3.05E-05	0.003168201
Lachnospiraceae UCG.004	Control vs. DMA	Group	DMA	-2.128084909	0.501263059	121	118	4.44E-05	0.004201328
Ruminococcaceae UCG.003	Control vs. DMA	Group	DMA	-2.154313827	0.511864349	121	101	5.12E-05	0.004434235
Lachnospiraceae UCG.003	Control vs. DMA	Group	DMA	-2.186784416	0.466126583	121	47	7.54E-06	0.001567387
Faecalibacterium	Control vs. DMA	Group	DMA	-2.225229658	0.499160927	121	121	1.93E-05	0.002716496
Parasutterella	Control vs. DMA	Group	DMA	-2.273289772	0.544253055	121	120	5.78E-05	0.004623246
Sutterella	Control vs. DMA	Group	DMA	-2.356101752	0.629675366	121	104	0.000286718	0.012989726
Coprococcus 2	Control vs. DMA	Group	DMA	-2.359932069	0.595005621	121	111	0.000127334	0.008828469
Alloprevotella	Control vs. DMA	Group	DMA	-2.531348135	0.687534133	121	78	0.000354262	0.013158291
Lachnospira	Control vs. DMA	Group	DMA	-2.657914462	0.585295277	121	119	1.39E-05	0.002404128
Paraprevotella	Control vs. DMA	Group	DMA	-2.789005314	0.689178701	121	97	9.44E-05	0.007013376
Prevotella 9	Control vs. DMA	Group	DMA	-3.178702046	0.939283448	121	116	0.00097701	0.022579781
Pseudomonas	Control vs. T2D	Group	T2D	4.939384931	0.930700458	108	90	6.52E-07	0.000118265
Enterococcus	Control vs. T2D	Group	T2D	3.021732366	0.570752113	108	92	6.89E-07	0.000118265
Allobaculum	Control vs. T2D	Group	T2D	2.81269656	0.537711663	108	43	9.04E-07	0.000132963
Comamonas	Control vs. T2D	Group	T2D	2.418887645	0.549815778	108	79	2.67E-05	0.00144927
Lactobacillus	Control vs. T2D	Group	T2D	2.367938603	0.480205676	108	108	3.18E-06	0.000273057
Sarcina	Control vs. T2D	Group	T2D	2.203292307	0.622248228	108	56	0.000602675	0.013494687
Stenotrophomonas	Control vs. T2D	Group	T2D	2.081052815	0.387964328	108	44	5.10E-07	0.000118265
Escherichia.Shigella	Control vs. T2D	Group	T2D	1.903095818	0.306008661	108	108	1.11E-08	5.72E-06
Lampropedia	Control vs. T2D	Group	T2D	1.898566303	0.378267235	108	42	2.21E-06	0.000227365
Pseudochrobactrum	Control vs. T2D	Group	T2D	1.82736702	0.328923887	108	37	2.22E-07	7.61E-05
Peptoclostridium	Control vs. T2D	Group	T2D	1.677631874	0.5659995	108	79	0.003780114	0.053779335
Hafnia	Control vs. T2D	Group	T2D	1.605305321	0.424470968	108	27	0.000262577	0.007800113
Aeromonas	Control vs. T2D	Group	T2D	1.585884351	0.340487371	108	27	9.67E-06	0.000601092
Eubacterium	Control vs. T2D	Group	T2D	1.581346842	0.383539367	108	58	7.63E-05	0.003024003
Psychrobacter	Control vs. T2D	Group	T2D	1.522673327	0.363733752	108	22	6.03E-05	0.002524935
Fusobacterium	Control vs. T2D	Group	T2D	1.406307612	0.613339821	108	90	0.023908142	0.220540762
Sporosarcina	Control vs. T2D	Group	T2D	1.387393638	0.270801436	108	34	1.43E-06	0.000183593
Acinetobacter	Control vs. T2D	Group	T2D	1.353079866	0.499153796	108	94	0.007877841	0.096319461
Alcaligenes	Control vs. T2D	Group	T2D	1.330620471	0.362592578	108	23	0.000388231	0.009996959
Corynebacterium 1	Control vs. T2D	Group	T2D	1.320625107	0.362146475	108	39	0.000420362	0.010560316
Eisenbergiella	Control vs. T2D	Group	T2D	1.292465785	0.342218216	108	71	0.000267395	0.007800113
Rothia	Control vs. T2D	Group	T2D	1.290603435	0.395275939	108	91	0.001490429	0.025498267
Acidovorax	Control vs. T2D	Group	T2D	1.238536061	0.266273535	108	27	9.92E-06	0.000601092
Luteibacter	Control vs. T2D	Group	T2D	1.224956101	0.265131402	108	20	1.12E-05	0.000642929
Arthrobacter	Control vs. T2D	Group	T2D	1.205465249	0.437577881	108	65	0.006954834	0.086306974
Photobacterium	Control vs. T2D	Group	T2D	1.072545927	0.296307623	108	20	0.000461078	0.01130739
Delftia	Control vs. T2D	Group	T2D	1.066514717	0.35400898	108	40	0.003265846	0.048054592
Eggerthella	Control vs. T2D	Group	T2D	1.055042897	0.28034162	108	106	0.000280198	0.007800113
Atopobium	Control vs. T2D	Group	T2D	1.027318138	0.290948806	108	49	0.000623183	0.013554407
Pelomonas	Control vs. T2D	Group	T2D	0.996430793	0.236072785	108	26	5.29E-05	0.002477097
Helicobacter	Control vs. T2D	Group	T2D	0.989890692	0.428785988	108	11	0.022983931	0.21694052
Mycoplasma	Control vs. T2D	Group	T2D	0.986193449	0.261120828	108	14	0.000267357	0.007800113
Trichococcus	Control vs. T2D	Group	T2D	0.952516983	0.373191313	108	100	0.012181768	0.136382838
X.Eubacterium. brachy group	Control vs. T2D	Group	T2D	0.949943626	0.252086258	108	45	0.00027541	0.007800113
Bifidobacterium	Control vs. T2D	Group	T2D	0.928408324	0.410755223	108	108	0.025930872	0.231908846
Variovorax	Control vs. T2D	Group	T2D	0.909422519	0.292763707	108	28	0.002453368	0.038287415
Variibacter	Control vs. T2D	Group	T2D	0.902717728	0.363525886	108	28	0.014649982	0.158836648
Gemmobacter	Control vs. T2D	Group	T2D	0.875525229	0.264488994	108	18	0.001289328	0.023517254
Vibrio	Control vs. T2D	Group	T2D	0.840412466	0.276943767	108	21	0.003056018	0.045618816
Pediococcus	Control vs. T2D	Group	T2D	0.836737988	0.298156987	108	22	0.00600242	0.07632707
Gemella	Control vs. T2D	Group	T2D	0.816719661	0.361805406	108	96	0.026117889	0.231908846
Blautia	Control vs. T2D	Group	T2D	0.80808337	0.278774504	108	108	0.004588063	0.061372796
Turicibacter	Control vs. T2D	Group	T2D	0.777126989	0.335620547	108	106	0.022588113	0.21694052
Sorangium	Control vs. T2D	Group	T2D	0.675134768	0.239883349	108	12	0.005864634	0.07632707
Phenylobacterium	Control vs. T2D	Group	T2D	0.667807867	0.209738496	108	21	0.001926528	0.031005054
Sinomonas	Control vs. T2D	Group	T2D	0.654847066	0.183944979	108	20	0.000564909	0.012930134
Steroidobacter	Control vs. T2D	Group	T2D	0.644131149	0.222011939	108	14	0.004552591	0.061372796
Parvimonas	Control vs. T2D	Group	T2D	0.641895654	0.283286189	108	22	0.025570521	0.231908846
Cloacibacillus	Control vs. T2D	Group	T2D	0.564787308	0.202849493	108	20	0.006395416	0.080332658
Paenochrobactrum	Control vs. T2D	Group	T2D	0.53728026	0.162734707	108	13	0.001325875	0.023517254
Macrococcus	Control vs. T2D	Group	T2D	0.503489951	0.167919657	108	11	0.003409619	0.049463488
GKS98 freshwater group	Control vs. T2D	Group	T2D	0.318782319	0.133287836	108	12	0.018603353	0.187857387
CL500.29 marine group	Control vs. T2D	Group	T2D	-0.573065623	0.231498099	108	13	0.014953376	0.160437262
Pyramidobacter	Control vs. T2D	Group	T2D	-0.603266375	0.259152182	108	51	0.021894841	0.214777963
Howardella	Control vs. T2D	Group	T2D	-0.851506367	0.224362598	108	29	0.000250527	0.007800113
Erysipelotrichaceae UCG.003	Control vs. T2D	Group	T2D	-0.921157167	0.342557468	108	107	0.008371333	0.09910889
Lachnospiraceae NK4A136 group	Control vs. T2D	Group	T2D	-0.931033439	0.285508732	108	108	0.001510092	0.025498267
Bacteroides	Control vs. T2D	Group	T2D	-0.948329836	0.320247034	108	108	0.003811545	0.053779335
Prevotellaceae UCG.001	Control vs. T2D	Group	T2D	-0.98745328	0.299281099	108	22	0.001335093	0.023517254
Lachnospiraceae ND3007 group	Control vs. T2D	Group	T2D	-1.044160588	0.337401005	108	106	0.002542827	0.039091216
Allisonella	Control vs. T2D	Group	T2D	-1.084582958	0.442813728	108	86	0.016017932	0.170087321
Gaiella	Control vs. T2D	Group	T2D	-1.153727219	0.347150213	108	28	0.001235671	0.023517254
Holdemania	Control vs. T2D	Group	T2D	-1.204240821	0.361167771	108	97	0.001192958	0.023253939

X.Eubacterium. ventriosum group	Control vs. T2D	Group	T2D	-1.220694432	0.319919345	108	107	0.00023309	0.007800113
Ruminococcus 1	Control vs. T2D	Group	T2D	-1.244925659	0.407987717	108	108	0.002903856	0.043984883
Faecalibacterium	Control vs. T2D	Group	T2D	-1.276007365	0.369659586	108	108	0.000811588	0.017059909
Parasutterella	Control vs. T2D	Group	T2D	-1.280415549	0.472904042	108	107	0.007948693	0.096319461
Family XIII UCG.001	Control vs. T2D	Group	T2D	-1.319668736	0.369538079	108	78	0.000544036	0.012735379
Ruminiclostridium	Control vs. T2D	Group	T2D	-1.347518461	0.285827997	108	107	7.70E-06	0.000566644
Ruminococcaceae UCG.003	Control vs. T2D	Group	T2D	-1.393997848	0.405808498	108	101	0.000857798	0.017670649
Ruminiclostridium 9	Control vs. T2D	Group	T2D	-1.402968948	0.48042921	108	93	0.004305546	0.059129492
Coprococcus 2	Control vs. T2D	Group	T2D	-1.427562751	0.548570332	108	99	0.010637363	0.120400921
Dialister	Control vs. T2D	Group	T2D	-1.437299178	0.434698148	108	107	0.0013053	0.023517254
Phascolarctobacterium	Control vs. T2D	Group	T2D	-1.503505769	0.471624935	108	108	0.001902932	0.031005054
Sutterella	Control vs. T2D	Group	T2D	-1.608949824	0.573107339	108	102	0.005984357	0.07632707
Lachnospiraceae UCG.004	Control vs. T2D	Group	T2D	-1.610073419	0.39634366	108	107	9.56E-05	0.003646106
Haemophilus	Control vs. T2D	Group	T2D	-1.628740678	0.443743708	108	107	0.000387276	0.009996959
Castellaniella	Control vs. T2D	Group	T2D	-1.711185939	0.460861203	108	47	0.000334158	0.009057442
Lachnospiraceae UCG.003	Control vs. T2D	Group	T2D	-1.734904647	0.436632246	108	53	0.000132349	0.004868558
Lachnospiraceae UCG.001	Control vs. T2D	Group	T2D	-1.753616464	0.419343795	108	104	6.13E-05	0.002524935
Lachnospiraceae UCG.005	Control vs. T2D	Group	T2D	-1.792565426	0.543756711	108	85	0.001347105	0.023517254
Ruminiclostridium 6	Control vs. T2D	Group	T2D	-1.806529228	0.541953451	108	94	0.001196562	0.023253939
Incertae Sedis	Control vs. T2D	Group	T2D	-1.851182179	0.379573501	108	108	3.98E-06	0.000314958
Butyricimonas	Control vs. T2D	Group	T2D	-1.935869024	0.462646657	108	90	6.07E-05	0.002524935
Lachnospiraceae UCG.010	Control vs. T2D	Group	T2D	-1.988999461	0.390730851	108	74	1.64E-06	0.000187384
Prevotellaceae Ga6A1 group	Control vs. T2D	Group	T2D	-2.030427927	0.432925268	108	25	8.50E-06	0.000583467
Holdemanella	Control vs. T2D	Group	T2D	-2.038782474	0.629327536	108	97	0.001616247	0.026850552
X.Eubacterium. ruminantium group	Control vs. T2D	Group	T2D	-2.129019203	0.499539198	108	106	4.53E-05	0.002221138
Prevotella 9	Control vs. T2D	Group	T2D	-2.226656414	0.712294583	108	105	0.002308367	0.036578746
Barnesiella	Control vs. T2D	Group	T2D	-2.246200505	0.522871616	108	98	3.98E-05	0.002049705
Paraprevotella	Control vs. T2D	Group	T2D	-2.268956987	0.577237633	108	97	0.000154463	0.005486105
Prevotella 2	Control vs. T2D	Group	T2D	-2.371904412	0.672516784	108	84	0.000631662	0.013554407
Mitsuokella	Control vs. T2D	Group	T2D	-2.541566648	0.670283748	108	61	0.000253592	0.007800113
Lachnospira	Control vs. T2D	Group	T2D	-2.748191048	0.421848605	108	107	2.80E-09	2.89E-06
Alloprevotella	Control vs. T2D	Group	T2D	-2.953103287	0.595005713	108	66	2.79E-06	0.000260864
Faecalibacterium	T2D vs. DMA	Group	DMA	-1.318823923	0.456938425	129	129	0.004606548	0.241543424
Ruminococcaceae NK4A214 group	T2D vs. DMA	Group	DMA	-1.129775112	0.393525186	129	128	0.004820627	0.241543424
Hafnia	T2D vs. DMA	Group	DMA	-1.108978032	0.386403153	129	37	0.004833588	0.241543424
Prevotellaceae NK3B31 group	T2D vs. DMA	Group	DMA	-1.005028004	0.354912332	129	37	0.00541012	0.249090926
Sporosarcina	T2D vs. DMA	Group	DMA	-0.869184037	0.285896058	129	54	0.002889227	0.223947421
Succinivibrio	T2D vs. DMA	Group	DMA	1.195115003	0.366308441	129	20	0.001429188	0.223947421

Table S5 Differences in bacterial species among healthy controls, T2D, and DMA revealed by MaAsLin2.

feature	Compare	metadata	value	coef	stderr	N	N.not.0	pval	qval
Lactobacillus acidophilus	Control vs. DMA	Group	DMA	4.810412869	0.714845902	121	71	7.00E-10	2.80E-07
Enterococcus faecalis	Control vs. DMA	Group	DMA	3.451096301	0.677466243	121	100	1.39E-06	0.00014619
Pseudomonas fluorescens	Control vs. DMA	Group	DMA	2.964754461	0.974196148	121	82	0.002900347	0.051104447
Eubacterium sp. ARC.2	Control vs. DMA	Group	DMA	2.362944122	0.464984959	121	65	1.46E-06	0.00014619
Lampropedia sp. 13Bin	Control vs. DMA	Group	DMA	1.766102853	0.477263853	121	45	0.000331743	0.013489331
Alcaligenes sp. OO4	Control vs. DMA	Group	DMA	1.572452431	0.490645921	121	16	0.001749216	0.038871462
Variovorax soli	Control vs. DMA	Group	DMA	1.510825825	0.403561165	121	50	0.00028473	0.013489331
bacterium BM0331	Control vs. DMA	Group	DMA	1.495999163	0.451299033	121	121	0.001226865	0.030671637
Klebsiella pneumoniae	Control vs. DMA	Group	DMA	1.435160724	0.525165252	121	121	0.007272583	0.083115233
Megasphaera sp. BS.4	Control vs. DMA	Group	DMA	1.375940396	0.57617848	121	50	0.018567246	0.148810053
Synergistetes bacterium MFA2	Control vs. DMA	Group	DMA	1.192358406	0.322624558	121	25	0.000337233	0.013489331
Bacillus megaterium	Control vs. DMA	Group	DMA	1.114943721	0.373231496	121	43	0.003441146	0.051104447
Vibrio anguillarum	Control vs. DMA	Group	DMA	0.87842038	0.410952901	121	38	0.034673315	0.220148034
Morganella morganii subsp. sibonii	Control vs. DMA	Group	DMA	0.821488218	0.338559789	121	22	0.016801855	0.140015456
Hafnia alvei	Control vs. DMA	Group	DMA	0.763913715	0.22748267	121	14	0.001064762	0.030421784
Streptococcus mutans	Control vs. DMA	Group	DMA	0.754110674	0.299170954	121	42	0.013083805	0.118943679
Sporosarcina newyorkensis	Control vs. DMA	Group	DMA	0.686865342	0.269024494	121	24	0.011983949	0.111478596
Mogibacterium sp. CM96	Control vs. DMA	Group	DMA	0.661313822	0.282529386	121	48	0.020971095	0.158272412
Staphylococcus equorum subsp. equorum	Control vs. DMA	Group	DMA	0.528753886	0.254547781	121	17	0.040007213	0.235336548
Dyella sp. CO69May	Control vs. DMA	Group	DMA	0.429157255	0.143315863	121	15	0.00336662	0.051104447
Unassigned	Control vs. DMA	Group	DMA	-0.335482276	0.112168222	121	121	0.003403624	0.051104447
Brevundimonas naejangsanensis	Control vs. DMA	Group	DMA	-0.463838094	0.219694155	121	23	0.036913187	0.223716287
Bacteroides sp. DSM 12148	Control vs. DMA	Group	DMA	-1.042608211	0.3782737	121	58	0.006801499	0.080612029
Parabacteroides distasonis	Control vs. DMA	Group	DMA	-1.620165265	0.604092561	121	109	0.008396379	0.090771664
Parabacteroides sp. D25	Control vs. DMA	Group	DMA	-1.633275455	0.324908636	121	30	1.85E-06	0.000147959
butyrate.producing bacterium L2.12	Control vs. DMA	Group	DMA	-1.651276946	0.599669994	121	111	0.006852022	0.080612029
Desulfovibrio piger	Control vs. DMA	Group	DMA	-1.729601798	0.339510951	121	25	1.38E-06	0.00014619
Weissella confusa	Control vs. DMA	Group	DMA	-1.747729879	0.572296579	121	86	0.00280725	0.051104447
Elbe River snow isolate Iso14	Control vs. DMA	Group	DMA	-1.861639293	0.423224677	121	43	2.45E-05	0.001630247
endophytic bacterium 8.2011.	Control vs. DMA	Group	DMA	-2.151310686	0.647138456	121	76	0.001189506	0.030671637
Bacteroides coprocola DSM 17136	Control vs. DMA	Group	DMA	-2.613274595	0.852491795	121	102	0.00270888	0.051104447
Pseudomonas fluorescens	Control vs. T2D	Group	T2D	5.473831458	1.015861029	108	62	4.59E-07	8.96E-05
Enterococcus faecalis	Control vs. T2D	Group	T2D	2.798842675	0.57643149	108	85	4.34E-06	0.000241935
Lactobacillus acidophilus	Control vs. T2D	Group	T2D	2.670218665	0.52212358	108	58	1.48E-06	0.000144491
bacterium BM0331	Control vs. T2D	Group	T2D	1.903095818	0.306008661	108	108	1.11E-08	4.33E-06
Lampropedia sp. 13Bin	Control vs. T2D	Group	T2D	1.898566303	0.378267235	108	42	2.21E-06	0.000151054
Hafnia alvei	Control vs. T2D	Group	T2D	1.605305321	0.424470968	108	27	0.000262577	0.007314634
Eubacterium sp. ARC.2	Control vs. T2D	Group	T2D	1.581346842	0.383539367	108	58	7.63E-05	0.002977029
Acinetobacter sp. IrT.R5M2.138	Control vs. T2D	Group	T2D	1.557256244	0.381577769	108	57	8.92E-05	0.003161726
Lactobacillus fermentum	Control vs. T2D	Group	T2D	1.5538761	0.533051502	108	49	0.004372014	0.055424764
Sporosarcina newyorkensis	Control vs. T2D	Group	T2D	1.387393638	0.270801436	108	34	1.43E-06	0.000144491
Streptococcus mutans	Control vs. T2D	Group	T2D	1.382436948	0.312242153	108	41	2.40E-05	0.001039291
Dyella sp. CO69May	Control vs. T2D	Group	T2D	1.224956101	0.265131402	108	20	1.12E-05	0.000547738
Arthrobacter sp. KFC.41	Control vs. T2D	Group	T2D	1.205465249	0.437577881	108	65	0.006954834	0.08219349
Bacillus megaterium	Control vs. T2D	Group	T2D	1.175800245	0.341322057	108	38	0.000830637	0.01704992
Alcaligenes sp. OO4	Control vs. T2D	Group	T2D	1.167761019	0.323612904	108	22	0.000479057	0.010379575
Eggerthella sp. E1	Control vs. T2D	Group	T2D	1.029641886	0.281353385	108	106	0.000402045	0.009223382
Pseudomonas aeruginosa	Control vs. T2D	Group	T2D	0.997980464	0.366977471	108	38	0.007687162	0.088176271
Streptococcus sp. oral taxon G59	Control vs. T2D	Group	T2D	0.997590912	0.397662067	108	107	0.013694914	0.140553067
Bacillus thuringiensis	Control vs. T2D	Group	T2D	0.966332769	0.36100113	108	33	0.008662187	0.096521518
Ruminococcus gnavus CC55 001C	Control vs. T2D	Group	T2D	0.935001069	0.402479229	108	106	0.022158721	0.200974442
Variovorax soli	Control vs. T2D	Group	T2D	0.909422519	0.292763707	108	28	0.002453368	0.038272546
Atopobium parvulum	Control vs. T2D	Group	T2D	0.902536999	0.283076855	108	49	0.001900638	0.032228207
Corynebacterium mucifaciens	Control vs. T2D	Group	T2D	0.866682547	0.288868455	108	27	0.003390471	0.047491123
Vibrio anguillarum	Control vs. T2D	Group	T2D	0.840412466	0.276943767	108	21	0.003056018	0.045840266
Pseudomonas caeni	Control vs. T2D	Group	T2D	0.814647192	0.262022028	108	14	0.002432682	0.038272546
Synergistetes bacterium MFA2	Control vs. T2D	Group	T2D	0.564787308	0.202849493	108	20	0.006395416	0.077944127
Macrococcus equipercicus	Control vs. T2D	Group	T2D	0.503489951	0.167919657	108	11	0.003409619	0.047491123
Unassigned	Control vs. T2D	Group	T2D	-0.302305557	0.075741616	108	108	0.000124003	0.004030112
Mitsuokella jalaludinii	Control vs. T2D	Group	T2D	-0.827916362	0.353351311	108	24	0.021067932	0.195630799
Holdemania filiformis DSM 12042	Control vs. T2D	Group	T2D	-1.173249284	0.350818337	108	84	0.001154813	0.022518861
Desulfovibrio piger	Control vs. T2D	Group	T2D	-1.274216198	0.345044594	108	27	0.000358335	0.008734426
butyrate.producing bacterium L2.12	Control vs. T2D	Group	T2D	-1.471972082	0.389162996	108	105	0.000262107	0.007314634
Parabacteroides sp. D25	Control vs. T2D	Group	T2D	-1.522611725	0.304109998	108	32	2.32E-06	0.000151054
endophytic bacterium 8.2011.	Control vs. T2D	Group	T2D	-1.603970636	0.550725193	108	67	0.004405558	0.055424764
Elbe River snow isolate Iso14	Control vs. T2D	Group	T2D	-1.711185939	0.460861203	108	47	0.000334158	0.00868811
Bacteroides coprocola DSM 17136	Control vs. T2D	Group	T2D	-2.209515395	0.672253403	108	103	0.001390617	0.025825737
Parabacteroides distasonis	DMA vs. T2D	Group	DMA	-1.406500823	0.497752631	129	116	0.00550708	0.219556955
Hafnia alvei	DMA vs. T2D	Group	DMA	-1.108978032	0.386403153	129	37	0.004833588	0.219556955
Sporosarcina newyorkensis	DMA vs. T2D	Group	DMA	-0.869184037	0.285896058	129	54	0.002889227	0.197430495
Lactobacillus acidophilus	DMA vs. T2D	Group	DMA	1.895404743	0.586866306	129	105	0.001589502	0.197430495

Table S7 Differences in fungal orders among healthy controls, T2D, and DMA revealed by MaAsLin2.

feature	Compare	metadata	value	coef	stderr	N	N.not.0	pval	qval
Malasseziales	Control vs. DMA	Group	DMA	-4.33036	0.622651	121	104	2.29E-10	1.43E-08
Neocallimastigales	Control vs. DMA	Group	DMA	-3.11452	0.382855	121	37	5.40E-13	6.75E-11
Capnodiales	Control vs. DMA	Group	DMA	-2.05629	0.481095	121	64	3.98E-05	0.001243
Polyporales	Control vs. DMA	Group	DMA	-2.02234	0.547711	121	60	0.000341	0.008534
Chaetothyriales	Control vs. DMA	Group	DMA	-1.95835	0.426923	121	57	1.15E-05	0.00048
Pleosporales	Control vs. DMA	Group	DMA	-1.72321	0.547316	121	88	0.002091	0.02614
Sordariales	Control vs. DMA	Group	DMA	-1.64115	0.487097	121	52	0.001026	0.016751
Wallemiales	Control vs. DMA	Group	DMA	-1.5447	0.460274	121	84	0.001072	0.016751
Tremellales	Control vs. DMA	Group	DMA	-1.36086	0.388341	121	65	0.000653	0.013612
Mortierellales	Control vs. DMA	Group	DMA	-1.16589	0.364973	121	54	0.001808	0.025113
Pezizales	Control vs. DMA	Group	DMA	-0.82836	0.387084	121	37	0.034467	0.239352
Ostropales	Control vs. DMA	Group	DMA	0.86966	0.381674	121	44	0.024541	0.191723
Cystobasidiales	Control vs. DMA	Group	DMA	1.086011	0.423033	121	47	0.011535	0.102993
Saccharomycetales	Control vs. DMA	Group	DMA	1.766156	0.578532	121	120	0.002816	0.031372
Malasseziales	Control vs. T2D	Group	T2D	-3.93789	0.580359	108	92	7.77E-10	5.05E-08
Neocallimastigales	Control vs. T2D	Group	T2D	-2.8121	0.351223	108	36	1.98E-12	2.57E-10
Pleosporales	Control vs. T2D	Group	T2D	-2.15395	0.44506	108	80	4.63E-06	0.000201
Capnodiales	Control vs. T2D	Group	T2D	-1.85887	0.487952	108	61	0.000238	0.006192
Chaetothyriales	Control vs. T2D	Group	T2D	-1.51197	0.378247	108	54	0.000121	0.003942
Ustilaginales	Control vs. T2D	Group	T2D	-0.54892	0.224192	108	11	0.016055	0.184782
Cystobasidiales	Control vs. T2D	Group	T2D	1.092309	0.358607	108	36	0.002952	0.054824
Auriculariales	Control vs. T2D	Group	T2D	1.177547	0.501527	108	34	0.020808	0.20808
Trichosporonales	Control vs. T2D	Group	T2D	1.285954	0.439342	108	106	0.00422	0.068579
Xylariales	T2D vs. DMA	Group	DMA	-1.21679	0.350915	129	38	0.000725	0.04589

Table S8 Differences in fungal families among healthy controls, T2D, and DMA revealed by MaAsLin2.

feature	Compare	metadata	value	coef	stderr	N	N.not.0	pval	qval
Malasseziaceae	Control vs. DMA	Group	DMA	-4.330362647	0.622651325	121	104	2.29E-10	2.29E-08
Neocallimastigaceae	Control vs. DMA	Group	DMA	-3.114520579	0.382854609	121	37	5.40E-13	1.08E-10
Herpotrichiellaceae	Control vs. DMA	Group	DMA	-1.87612241	0.397055995	121	50	6.56E-06	0.000328063
Glomeraceae	Control vs. DMA	Group	DMA	-1.853089875	0.350107558	121	40	5.84E-07	3.89E-05
Cladosporiaceae	Control vs. DMA	Group	DMA	-1.814237886	0.465254565	121	63	0.00016265	0.004647149
Polyporaceae	Control vs. DMA	Group	DMA	-1.732981767	0.388331851	121	34	1.90E-05	0.000759431
Wallemiaceae	Control vs. DMA	Group	DMA	-1.544695398	0.460273693	121	84	0.001072082	0.022637415
Chaetomiaceae	Control vs. DMA	Group	DMA	-1.427472247	0.427449036	121	37	0.001131871	0.022637415
Phanerochaetaceae	Control vs. DMA	Group	DMA	-1.218361293	0.308571099	121	30	0.000135974	0.004532469
Unassigned	Control vs. DMA	Group	DMA	-1.130091334	0.443535704	121	120	0.012155954	0.121139266
Mortierellaceae	Control vs. DMA	Group	DMA	-1.070024449	0.358024173	121	52	0.003426336	0.04568448
Bulleribasidiaceae	Control vs. DMA	Group	DMA	-0.945098545	0.294287497	121	45	0.001712689	0.031139799
Phaeosphaeriaceae	Control vs. DMA	Group	DMA	-0.835561336	0.330111426	121	37	0.012719623	0.121139266
V40	Control vs. DMA	Group	DMA	0.311356266	0.080950517	121	121	0.000197278	0.004931945
Graphidaceae	Control vs. DMA	Group	DMA	0.869660416	0.381674434	121	44	0.024540604	0.18877388
Cystobasidiaceae	Control vs. DMA	Group	DMA	1.086011087	0.42303253	121	47	0.011535256	0.121139266
Dipodascaceae	Control vs. DMA	Group	DMA	1.736180996	0.561370586	121	71	0.002489209	0.041486821
Debaryomycetaceae	Control vs. DMA	Group	DMA	1.931215462	0.820449139	121	92	0.020276862	0.168973852
Debaryomycetaceae	Control vs. T2D	Group	T2D	2.337153814	0.784440544	108	76	0.00361009	0.079095426
Auriculariaceae	Control vs. T2D	Group	T2D	1.308580336	0.475534622	108	27	0.007015333	0.116922214
Trichosporonaceae	Control vs. T2D	Group	T2D	1.297458152	0.439984126	108	106	0.003954771	0.079095426
Cystobasidiaceae	Control vs. T2D	Group	T2D	1.035057938	0.359608108	108	35	0.004871284	0.088568796
V40	Control vs. T2D	Group	T2D	0.285242301	0.061981144	108	108	1.21E-05	0.00048299
Phaeosphaeriaceae	Control vs. T2D	Group	T2D	-0.756442007	0.279214372	108	33	0.007912598	0.121732273
Unassigned	Control vs. T2D	Group	T2D	-0.788334602	0.297832894	108	108	0.00941158	0.13445114
Phanerochaetaceae	Control vs. T2D	Group	T2D	-1.005399424	0.290078699	108	29	0.000774452	0.022127187
Cladosporiaceae	Control vs. T2D	Group	T2D	-1.611961908	0.475114031	108	60	0.00098598	0.024649499
Polyporaceae	Control vs. T2D	Group	T2D	-1.632378043	0.388958444	108	34	5.79E-05	0.00193103
Herpotrichiellaceae	Control vs. T2D	Group	T2D	-1.650177297	0.346563501	108	47	6.37E-06	0.000318349
Glomeraceae	Control vs. T2D	Group	T2D	-1.923276961	0.326020915	108	41	4.79E-08	3.19E-06
Neocallimastigaceae	Control vs. T2D	Group	T2D	-2.812098018	0.351222724	108	36	1.98E-12	3.95E-10
Malasseziaceae	Control vs. T2D	Group	T2D	-3.93789125	0.580358513	108	92	7.77E-10	7.77E-08

Table S9 Differences in fungal genus among healthy controls, T2D, and DMA revealed by MaAsLin2.

feature	Compare	metadata	value	coef	stderr	N	N.not.0	pval	qval
Malassezia	Control vs. DMA	Group	DMA	-4.360216785	0.620516601	121	104	1.60E-10	1.30E-08
Pecoramyces	Control vs. DMA	Group	DMA	-2.030976844	0.275436052	121	33	2.77E-11	4.49E-09
Trametes	Control vs. DMA	Group	DMA	-1.943514353	0.357714092	121	28	3.14E-07	2.04E-05
Orpinomyces	Control vs. DMA	Group	DMA	-1.925806602	0.271889567	121	32	1.20E-10	1.30E-08
Cladosporium	Control vs. DMA	Group	DMA	-1.754006029	0.444064017	121	53	0.000135228	0.003995368
Exophiala	Control vs. DMA	Group	DMA	-1.746024716	0.365864553	121	44	5.40E-06	0.000250621
Wallemia	Control vs. DMA	Group	DMA	-1.544695398	0.460273693	121	84	0.001072082	0.026802042
Phanerochaete	Control vs. DMA	Group	DMA	-1.142658387	0.274383504	121	24	6.06E-05	0.002188166
Symmetrospora	Control vs. DMA	Group	DMA	-1.140134043	0.280838934	121	29	9.00E-05	0.002923972
Cyllamyces	Control vs. DMA	Group	DMA	-0.955741459	0.183601621	121	27	8.56E-07	4.64E-05
Hannaella	Control vs. DMA	Group	DMA	-0.930258603	0.23959573	121	25	0.000172927	0.004683449
Mortierella	Control vs. DMA	Group	DMA	-0.868027883	0.338711507	121	49	0.011676484	0.130857153
Caecomyces	Control vs. DMA	Group	DMA	-0.715361668	0.161768038	121	19	2.23E-05	0.000905623
Ceratobasidium	Control vs. DMA	Group	DMA	-0.615829852	0.225865941	121	16	0.007403084	0.100250097
Neosetophoma	Control vs. DMA	Group	DMA	-0.509209021	0.202147874	121	14	0.013142875	0.133482321
Glomus	Control vs. DMA	Group	DMA	-0.507105147	0.162454435	121	19	0.002275669	0.052828034
Rhizophagus	Control vs. DMA	Group	DMA	-0.485212898	0.159440002	121	19	0.002900801	0.058922521
Saccharomyces	Control vs. DMA	Group	DMA	0.513283832	0.166148551	121	18	0.002516006	0.054513466
Saccharomycopsis	Control vs. DMA	Group	DMA	0.702273556	0.252864465	121	14	0.006402517	0.09870611
Gomphillus	Control vs. DMA	Group	DMA	0.869660416	0.381674434	121	44	0.024540604	0.221547123
Xeromyces	Control vs. DMA	Group	DMA	1.001964644	0.39078329	121	21	0.011636661	0.130857153
Apiotrichum	Control vs. DMA	Group	DMA	1.089251669	0.396537795	121	35	0.006985356	0.09870611
Cystobasidium	Control vs. DMA	Group	DMA	1.115798754	0.40146197	121	46	0.006364523	0.09870611
Meyerozyma	Control vs. DMA	Group	DMA	2.32613557	0.834762376	121	83	0.006232894	0.09870611
Malassezia	Control vs. T2D	Group	T2D	-4.001219285	0.575232293	108	92	3.43E-10	2.70E-08
Talaromyces	Control vs. T2D	Group	T2D	-2.861264848	0.389206714	108	50	5.04E-11	5.29E-09
Pecoramyces	Control vs. T2D	Group	T2D	-1.989181807	0.253292511	108	33	4.24E-12	1.34E-09
Orpinomyces	Control vs. T2D	Group	T2D	-1.893282093	0.25001508	108	32	1.70E-11	2.68E-09
Cladosporium	Control vs. T2D	Group	T2D	-1.838605554	0.464273304	108	51	0.000138845	0.004859569
Trametes	Control vs. T2D	Group	T2D	-1.612130667	0.376756398	108	31	4.25E-05	0.001671486
Exophiala	Control vs. T2D	Group	T2D	-1.458953118	0.322409336	108	42	1.64E-05	0.00084162
Cyllamyces	Control vs. T2D	Group	T2D	-0.9188926	0.167559891	108	27	3.03E-07	1.91E-05
Phanerochaete	Control vs. T2D	Group	T2D	-0.88065711	0.28794896	108	27	0.002842503	0.063956328
Symmetrospora	Control vs. T2D	Group	T2D	-0.738230393	0.287163144	108	32	0.011590692	0.135872036
Ceratobasidium	Control vs. T2D	Group	T2D	-0.657899463	0.189020748	108	17	0.00073768	0.023236923
Rhizophagus	Control vs. T2D	Group	T2D	-0.605368286	0.134789865	108	19	1.87E-05	0.00084162
Neosetophoma	Control vs. T2D	Group	T2D	-0.526433918	0.181783778	108	13	0.004625527	0.091065068
Naganishia	Control vs. T2D	Group	T2D	-0.492555517	0.183226559	108	11	0.008390687	0.114915929
Glomus	Control vs. T2D	Group	T2D	-0.475664507	0.168402906	108	20	0.005695565	0.094426469
Caecomyces	Control vs. T2D	Group	T2D	-0.461124539	0.139587542	108	18	0.001317761	0.033632903
Iodophanus	Control vs. T2D	Group	T2D	-0.391145831	0.136323332	108	11	0.005001868	0.092681671
Hanseniaspora	Control vs. T2D	Group	T2D	0.4209319	0.160818012	108	11	0.010206541	0.133960855
Mucor	Control vs. T2D	Group	T2D	0.50302836	0.194071101	108	13	0.010941979	0.135872036
Vanrija	Control vs. T2D	Group	T2D	1.014924227	0.30873989	108	15	0.001388025	0.033632903
Cystobasidium	Control vs. T2D	Group	T2D	1.032489138	0.34614188	108	34	0.003572807	0.07502895
Apiotrichum	Control vs. T2D	Group	T2D	1.171754946	0.413058612	108	26	0.005497704	0.094426469
Auricularia	Control vs. T2D	Group	T2D	1.308580336	0.475534622	108	27	0.007015333	0.105229992
Penicillium	Control vs. T2D	Group	T2D	1.798441707	0.528674531	108	78	0.000957288	0.027413245
Meyerozyma	Control vs. T2D	Group	T2D	2.27671746	0.832791977	108	61	0.007381661	0.105691967

Table S10 Differences in fungal species among healthy controls, T2D, and DMA revealed by MaAsLin2.

feature	Compare	metadata	value	coef	stderr	N	N.not.0	pval	qval
Meyerozyma guilliermondii	Control vs. DMA	Group	DMA	2.655287	0.860329	121	75	0.002539	0.074903
Xeromyces bisporus	Control vs. DMA	Group	DMA	1.001965	0.390783	121	21	0.011637	0.214551
Saccharomycopsis fibuligera	Control vs. DMA	Group	DMA	0.702274	0.252864	121	14	0.006403	0.125916
Saccharomyces cariocanus	Control vs. DMA	Group	DMA	0.513284	0.166149	121	18	0.002516	0.074903
Caecomyces churrovis	Control vs. DMA	Group	DMA	-0.38619	0.134414	121	15	0.004841	0.109858
Hannaella zeae	Control vs. DMA	Group	DMA	-0.40563	0.138747	121	14	0.004169	0.102488
Hannaella sinensis	Control vs. DMA	Group	DMA	-0.4672	0.165993	121	14	0.005747	0.121102
Symmetrospora oryzicola	Control vs. DMA	Group	DMA	-1.06303	0.256873	121	25	6.69E-05	0.00282
Cutaneotrichosporon debeurmannianum	Control vs. DMA	Group	DMA	-1.09853	0.238564	121	22	1.07E-05	0.000527
Cutaneotrichosporon curvatum	Control vs. DMA	Group	DMA	-1.6887	0.531952	121	109	0.001926	0.071025
Talaromyces funiculosus	Control vs. DMA	Group	DMA	-1.79416	0.270659	121	31	1.15E-09	8.46E-08
Talaromyces rugulosus	Control vs. DMA	Group	DMA	-1.84818	0.275259	121	33	7.54E-10	7.41E-08
Trametes trogii	Control vs. DMA	Group	DMA	-1.94351	0.357714	121	28	3.14E-07	1.85E-05
Pecoramyces ruminantium	Control vs. DMA	Group	DMA	-2.03098	0.275436	121	33	2.77E-11	4.08E-09
Malassezia globosa	Control vs. DMA	Group	DMA	-4.96394	0.605322	121	47	3.83E-13	1.13E-10
Meyerozyma guilliermondii	Control vs. T2D	Group	T2D	1.607106	0.660419	108	50	0.016695	0.222593
Auricularia auricula.judae	Control vs. T2D	Group	T2D	1.250605	0.46023	108	26	0.007733	0.119377
Cutaneotrichosporon cutaneum	Control vs. T2D	Group	T2D	1.18964	0.330437	108	23	0.000493	0.016897
Apiotrichum domesticum	Control vs. T2D	Group	T2D	1.094835	0.384701	108	20	0.005354	0.098834
Vanrija humicola	Control vs. T2D	Group	T2D	1.014924	0.30874	108	15	0.001388	0.037014
Hannaella sinensis	Control vs. T2D	Group	T2D	-0.50057	0.1545	108	15	0.001615	0.038749
Talaromyces purpureogenus	Control vs. T2D	Group	T2D	-0.77349	0.231349	108	12	0.001158	0.034748
Symmetrospora oryzicola	Control vs. T2D	Group	T2D	-0.79392	0.255037	108	27	0.002404	0.048071
Cutaneotrichosporon debeurmannianum	Control vs. T2D	Group	T2D	-1.0752	0.218705	108	22	3.38E-06	0.000135
Talaromyces funiculosus	Control vs. T2D	Group	T2D	-1.53103	0.272344	108	34	1.66E-07	9.93E-06
Trametes trogii	Control vs. T2D	Group	T2D	-1.75934	0.341298	108	30	1.25E-06	5.99E-05
Talaromyces rugulosus	Control vs. T2D	Group	T2D	-1.85338	0.252591	108	33	5.39E-11	4.32E-09
Pecoramyces ruminantium	Control vs. T2D	Group	T2D	-1.98918	0.253293	108	33	4.24E-12	5.09E-10
Malassezia globosa	Control vs. T2D	Group	T2D	-4.72916	0.558428	108	46	1.95E-13	4.67E-11

Table S11 The integrated signatures consisting of mycobiome as the optimal marker set between T2D, DM and healthy controls.

Name	Mean Decrease Gini	Increase in group
Pecoramyces ruminantium	3.234659276	Decreased in T2D&DM (vs Control)
Meyerozyma guilliermondii	3.208551196	Increased in T2D&DM (vs Control)
Talaromyces funiculosus	3.117177857	Decreased in T2D&DM (vs Control)
Talaromyces rugulosus	3.114465942	Decreased in T2D&DM (vs Control)
Cutaneotrichosporon curvatum	2.619818596	None
Trametes trogii	2.269164524	Decreased in T2D&DM (vs Control)
Cutaneotrichosporon cutaneum	2.015129157	Decreased in T2D&DM (vs Control)
Candida parapsilosis	1.820724824	None
Fusarium oxysporum	1.795141699	None
Cutaneotrichosporon debeurmannianum	1.661439858	Decreased in T2D&DM (vs Control)
Aspergillus hiratsukae	1.651581622	Increased in T2D&DM (vs Control)
Candida albicans	1.57057918	None
Auricularia auricula.judae	1.491792105	Increased in T2D (vs Control)
Aspergillus penicillioides	1.469883162	None
Phlyctis speirea	1.445207289	None
Apiotrichum domesticum	1.437336135	Increased in T2D&DM (vs Control)
Aspergillus ruber	1.388562674	None
Gomphillus calycioides	1.331503935	None
Candida tropicalis	1.271996233	None
Vanrija humicola	1.126270065	Increased in T2D&DM (vs Control)
Symmetrospora oryzicola	1.122407148	Decreased in T2D&DM (vs Control)
Effuseotrichosporon vanderwaltii	1.057503822	Increased in DM (vs Control)
Cutaneotrichosporon jirovecii	1.008924127	None
Wallemia sebi	0.989871635	None
Saccharomyces cariocanus	0.987192543	Increased in T2D&DM (vs Control)
Kurtzmaniella quercitrusa	0.954432607	None
Tausonia pullulans	0.951260988	Increased in DM (vs Control)
Issatchenkia orientalis	0.94315019	Increased in DM (vs Control)
Aspergillus piperis	0.924917973	None
Yarrowia alimentaria	0.907959612	Increased in DM (vs Control)
Cystobasidium terricola	0.901938371	Increased in T2D&DM (vs Control)
Trichosporon asteroides	0.891248502	None
Penicillium salamorum	0.884839883	Decreased in T2D (vs Control)
Malassezia restricta	0.854066364	DEcreased in DM (vs Control)
Kazachstania bulderi	0.836215868	None
Mortierella globalpina	0.831165315	None
Xeromyces bisporus	0.801324278	Increased in DM (vs Control)
Wallemia canadensis	0.772275793	None
Wickerhamomyces anomalus	0.763300312	Increased in DM (vs Control)
Bullera alba	0.755192248	None
Aspergillus fumigatus	0.685515536	None
Nigrospora oryzae	0.679294585	Decreased in T2D&DM (vs Control)
Stellatospora terricola	0.670700952	Increased in T2D&DM (vs Control)
Sterigmatomyces halophilus	0.660569078	None
Kodamaea ohmeri	0.627543744	None
Hannaella zeae	0.627151461	Increased in DM (vs Control)
Ganoderma sichuanense	0.59316971	None
Pichia membranifaciens	0.591754129	Increased in T2D&DM (vs Control)
Wickerhamomyces onychis	0.564659193	None
Cystobasidium laryngis	0.539613927	Increased in T2D&DM (vs Control)

Table S12 The integrated signatures consisting of bacteriome as the optimal marker set between T2D, DM and healthy controls.

Name	Mean Decrease Gini	Change in group
Bacterium BM0331	2.639826162	Increased in T2D & DM(vs Control)
Enterococcus faecalis	2.428583482	Increased in T2D & DM(vs Control)
butyrate.producing bacterium L2.12	2.368104152	Decreased in T2D & DM(vs Control)
Eggerthella sp. E1	2.364517541	Increased in T2D & DM(vs Control)
Bacteroides coprocola DSM 17136	2.245925178	Decreased in T2D & DM(vs Control)
Pseudomonas fluorescens	2.102147352	Increased in T2D & DM(vs Control)
Eubacterium sp. ARC.2	2.093650445	Increased in T2D & DM(vs Control)
Klebsiella pneumoniae	1.949253731	Increased in & DM(vs Control)
Parabacteroides distasonis	1.893676611	Decreased in T2D & DM(vs Control)
Ruminococcus gnavus CC55 001C	1.879689444	Increased in T2D & DM(vs Control)
Elbe River snow isolate Iso14	1.771828141	Decreased in T2D & DM(vs Control)
Megasphaera elsdenii	1.719293506	None
Streptococcus sp. 2011 Oral MS H4	1.70108603	None
Streptococcus sp. oral taxon G59	1.68110418	Increased in T2D (vs Control)
Arthrobacter sp. KFC.41	1.647028325	Increased in T2D & DM(vs Control)
Sporosarcina newyorkensis	1.625282602	Increased in T2D & DM(vs Control)
Bacillus thuringiensis	1.597418969	Increased in T2D & DM(vs Control)
Bacteroides fragilis str. 3998T.B.3	1.560799	None
Variovorax soli	1.550287636	Increased in T2D & DM(vs Control)
Streptococcus gallolyticus subsp. pasteuriani	1.548784615	Increased in T2D & DM(vs Control)
endophytic bacterium 8.2011.	1.542015331	Decreased in T2D & DM(vs Control)
Ruminococcus sp. RLB3	1.537942589	None
Lampropedia sp. 13Bin	1.506269152	Increased in T2D & DM(vs Control)
Weissella confusa	1.499402774	Decreased in T2D & DM(vs Control)
Parabacteroides sp. D25	1.389745405	Decreased in T2D & DM(vs Control)
Lactococcus lactis	1.365347981	None
Lactobacillus fermentum	1.338087211	Increased in T2D & DM(vs Control)
Streptococcus mutans	1.333701021	Increased in T2D & DM(vs Control)
Bacillus megaterium	1.330032569	Increased in T2D & DM(vs Control)
Holdemania filiformis DSM 12042	1.329820044	Increased in T2D(vs Control)
Hafnia alvei	1.308786153	Increased in T2D & DM(vs Control)
Veillonella atypica	1.30382236	Decreased in DM(vs Control)
Pseudomonas aeruginosa	1.30379273	Increased in T2D & DM(vs Control)
Megasphaera sp. BS.4	1.249794327	None
Clostridium sp. MLG661	1.234376203	Increased in T2D(vs Control)
Bifidobacterium bifidum	1.119005918	None
Acinetobacter sp. IrT.R5M2.138	1.107092671	Increased in T2D (vs Control)
Atopobium parvulum	1.088257157	Increased in T2D & DM(vs Control)
Bacteroides sp. DSM 12148	1.072838362	Decreased in T2D & DM(vs Control)
Alcaligenes sp. OO4	1.045429687	Increased in T2D & DM(vs Control)
Gordonibacter pamelaeae	1.033578139	None
Megasphaera micronuciformis	1.016068336	None
Peptoclostridium difficile CD9	0.993597228	None
Diaphorobacter sp. BAB.3478	0.972798049	None
bacterium NLAE.zl.P36	0.955660123	Increased in DM(vs Control)
Eggerthella sinensis	0.951843268	Increased in T2D & DM(vs Control)
Synergistetes bacterium MFA2	0.929270974	Increased in T2D & DM(vs Control)
Corynebacterium mucifaciens	0.921190759	Increased in T2D(vs Control)
Dyella sp . CO69May	0.917239018	Increased in T2D(vs Control)
Lactobacillus sanfranciscensis	0.878010659	None

Table S13 The integrated signatures consisting of fungi and bacteria as the optimal marker set between T2D, DM and healthy controls.

Name	Mean Decrease Gini	Change in group
bacterium BM0331	2.697320216	Increased in T2D & DM(vs Control)
Pecoramyces ruminantium	2.674470069	Decreased in T2D & DM(vs Control)
Talaromyces rugulosus	2.459518989	Decreased in T2D & DM(vs Control)
Talaromyces funiculosus	2.358597916	Decreased in T2D & DM(vs Control)
Enterococcus faecalis	2.195534518	Increased in T2D & DM(vs Control)
Eggerthella sp. E1	2.145924206	Increased in T2D & DM(vs Control)
butyrate.producing bacterium L2.1	2.113772616	Decreased in T2D & DM(vs Control)
Klebsiella pneumoniae	2.038201889	Increased in & DM(vs Control)
Bacteroides coprocola DSM 17136	1.974397631	Decreased in T2D & DM(vs Control)
Trametes trogii	1.687219835	Decreased in T2D & DM(vs Control)
Meyerozyma guilliermondii	1.677757595	Increased in T2D & DM(vs Control)
Pseudomonas fluorescens	1.56987297	Increased in T2D & DM(vs Control)
Eubacterium sp. ARC.2	1.533843998	Increased in T2D & DM(vs Control)
Parabacteroides distasonis	1.523006957	Decreased in T2D & DM(vs Control)
Ruminococcus gnavus CC55 001C	1.450292372	Increased in T2D & DM(vs Control)
Streptococcus sp. oral taxon G59	1.413090224	Increased in T2D (vs Control)
Hafnia alvei	1.406083119	Increased in T2D & DM(vs Control)
Sporosarcina newyorkensis	1.399010641	Increased in T2D & DM(vs Control)
Elbe River snow isolate Iso14	1.337914417	Decreased in T2D & DM(vs Control)
Parabacteroides sp. D25	1.328099736	Decreased in T2D & DM(vs Control)
Megasphaera elsdenii	1.310273782	None
Streptococcus sp. 2011 Oral MS H	1.246609795	None
Bacteroides fragilis str. 3998T.B.3	1.209657801	None
Ruminococcus sp. RLB3	1.204136618	None
Cutaneotrichosporon debeurmannic	1.184974598	Decreased in T2D & DM(vs Control)
Arthrobacter sp. KFC.41	1.158337239	Increased in T2D & DM(vs Control)
Cutaneotrichosporon curvatum	1.145223948	None
Bacillus megaterium	1.105741359	Increased in T2D & DM(vs Control)
Streptococcus gallolyticus subsp. pc	1.095999192	Increased in T2D & DM(vs Control)
Holdemania filiformis DSM 12042	1.084248351	Increased in T2D(vs Control)
Lampropedia sp. 13Bin	1.044614636	Increased in T2D & DM(vs Control)
Veillonella atypica	1.028094239	Decreased in DM(vs Control)
Variovorax soli	1.006675141	Increased in T2D & DM(vs Control)
Acinetobacter sp. IrT.R5M2.138	0.973929085	Increased in T2D (vs Control)
endophytic bacterium 8.2011.	0.971767827	Decreased in T2D & DM(vs Control)
Weissella confusa	0.891493309	Decreased in T2D & DM(vs Control)
Bacteroides sp. DSM 12148	0.875750995	Decreased in T2D & DM(vs Control)
Lactobacillus fermentum	0.860268494	Increased in T2D & DM(vs Control)
Bacillus thuringiensis	0.81236684	Increased in T2D & DM(vs Control)
Streptococcus mutans	0.800093162	Increased in T2D & DM(vs Control)
Lactococcus lactis	0.779718017	None
Fusarium oxysporum	0.77530938	None
Candida parapsilosis	0.773536411	None
Bifidobacterium bifidum	0.770686475	None
Synergistetes bacterium MFA2	0.760957137	Increased in T2D & DM(vs Control)
Dyella sp. CO69May	0.755036609	Increased in T2D(vs Control)
Clostridium sp. MLG661	0.739872193	Increased in T2D(vs Control)
Desulfovibrio piger	0.726908701	Decreased in T2D & DM(vs Control)
Pseudomonas aeruginosa	0.719916942	Increased in T2D & DM(vs Control)
Atopobium parvulum	0.698714579	Increased in T2D & DM(vs Control)
